# Collagenous Extracellular Matrix Biomaterials for Tissue Engineering: Lessons from the Common Sea Urchin Tissue

**DOI:** 10.3390/ijms18050901

**Published:** 2017-04-25

**Authors:** Kheng Lim Goh, David F. Holmes

**Affiliations:** 1 Newcastle University Singapore, SIT Building at Nanyang Polytechnic, 172A Ang Mo Kio Avenue 8 #05-01, Singapore 567739, Singapore; 2 Newcastle University, School of Mechanical & Systems Engineering, Stephenson Building, Claremont Road, Newcastle upon Tyne NE1 7RU, UK; 3 Manchester University, Wellcome Trust Centre for Cell Matrix Research, B.3016 Michael Smith Building, Faculty of Life Sciences, Oxford Road, Manchester M13 9PT, UK; david.f.holmes@manchester.ac.uk

**Keywords:** mutable collagenous tissues, decellularised tissue, collagen fibril, elastic stress transfer, plastic stress transfer, fracture, fibril taper, fibril diameter, fibril-fibril interactions

## Abstract

Scaffolds for tissue engineering application may be made from a collagenous extracellular matrix (ECM) of connective tissues because the ECM can mimic the functions of the target tissue. The primary sources of collagenous ECM material are calf skin and bone. However, these sources are associated with the risk of having bovine spongiform encephalopathy or transmissible spongiform encephalopathy. Alternative sources for collagenous ECM materials may be derived from livestock, e.g., pigs, and from marine animals, e.g., sea urchins. Collagenous ECM of the sea urchin possesses structural features and mechanical properties that are similar to those of mammalian ones. However, even more intriguing is that some tissues such as the ligamentous catch apparatus can exhibit mutability, namely rapid reversible changes in the tissue mechanical properties. These tissues are known as mutable collagenous tissues (MCTs). The mutability of these tissues has been the subject of on-going investigations, covering the biochemistry, structural biology and mechanical properties of the collagenous components. Recent studies point to a nerve-control system for regulating the ECM macromolecules that are involved in the sliding action of collagen fibrils in the MCT. This review discusses the key attributes of the structure and function of the ECM of the sea urchin ligaments that are related to the fibril-fibril sliding action—the focus is on the respective components within the hierarchical architecture of the tissue. In this context, structure refers to size, shape and separation distance of the ECM components while function is associated with mechanical properties e.g., strength and stiffness. For simplicity, the components that address the different length scale from the largest to the smallest are as follows: collagen fibres, collagen fibrils, interfibrillar matrix and collagen molecules. Application of recent theories of stress transfer and fracture mechanisms in fibre reinforced composites to a wide variety of collagen reinforcing (non-mutable) connective tissue, has allowed us to draw general conclusions concerning the mechanical response of the MCT at specific mechanical states, namely the stiff and complaint states. The intent of this review is to provide the latest insights, as well as identify technical challenges and opportunities, that may be useful for developing methods for effective mechanical support when adapting decellularised connective tissues from the sea urchin for tissue engineering or for the design of a synthetic analogue.

## 1. Introduction

**Figure 1:**
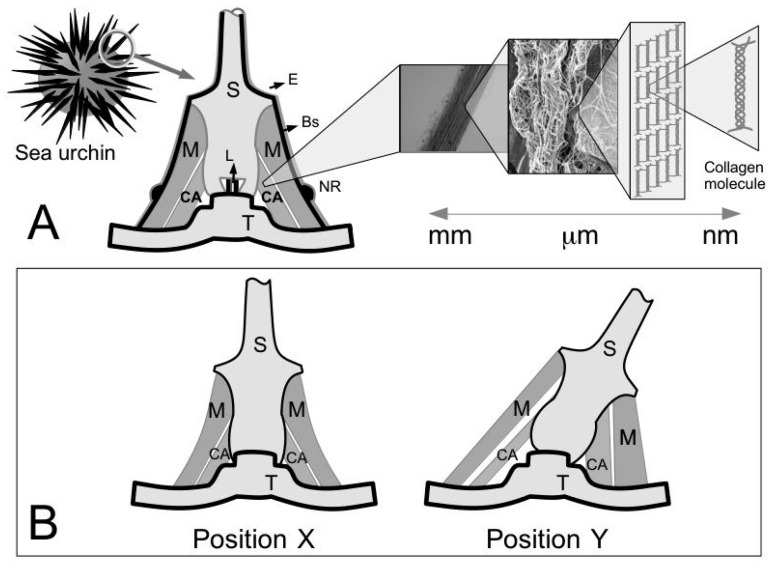
Sketches of the spine-test system of the sea urchin. (**A**) Cartoons of the sea urchin, represented by a sphere covered in spines, magnified view of the cross-section of the joint of the spine-test system and the hierarchical architecture of the catch apparatus (CA) tissue. The CA may be regarded as a ligamentous tissue as its ends are embedded in hard tissues of the spine and test; (**B**) Two positions, i.e., X and Y, of the spine. Symbols: S, spine; NR, nerve ring; Bs, basiepidermal nerve plexus; E, epidermis; L, central ligament; M, spine muscle; T, test. Adapted from Smith et al. [14], Hidaka et al. [15,16] and Motokawa and Fuchigami [17].

With regards to biomaterials for tissue engineering, the key areas that must be addressed are the cells, scaffolds and growth-stimulating signals [1,2,3]. The three areas are also collectively known as the tissue engineering triad [1,2,3]. In particular, scaffolds are intended to provide sites for cells attachment during the initial stages of the tissue engineering process—subsequently they serve as templates for enabling tissue materials to be generated onto the structure [3,4]. Polymeric biomaterials, such as chitosan, have been proposed for making scaffolds [5,6,7,8] but in order to satisfy the requirements of biocompatibility, the scaffold should ideally be made from the extracellular matrix (ECM) of the target tissue in its native state [3]. Where this is unavailable, the alternatives are bioengineered porous scaffolds [9], decellularized tissue from allogenic or xenogenic tissues [10,11], cell sheets with self-secreted ECM [12], and cell encapsulation in self-assembled hydrogel matrix [13]. A detailed discussion of the advantages and limitations of the different types of biological materials can be found elsewhere [3]. In this review, the focus is on the ECM derived from decellularized tissue (ECM-DT). A possible source for ECM-DTs is the connective tissue of the sea urchin (see sketch in Figure 1A), which belongs to a phylum of marine invertebrates called echinoderms. The discussion in this review is concerned with studies on the structure and mechanical properties of the ECM of the sea urchin’s connective tissue and the contribution of the findings from these studies to new insights for developing novel biomaterials for tissue engineering applications.

Echinoderms, such as sea urchin and starfish, are some of the most abundant multi-cellular animals in the marine world [18,19] and play a dominant role in the ecological chain [18,19]. In recent years, significant technological advancements have been achieved in the processing of tissue materials from marine animals for biomedical applications [20]. These biomedical applications range from post-surgical wound-assist healing to implants for tissue engineering and drug delivery [20,21]. With regards to marine sources, the connective tissue of echinoderms, such as the sea urchin, is a rich source of collagen which provides the structural support for the tissue [22,23]. Collagen is a protein molecule comprising three polypeptide alpha chains, organized in a triple-helical conformation [24,25,26,27,28,29,30,31]. There are at least twenty eight distinct types of collagen in vertebrates [25]. The major ones, namely type I, II and III, V and XI are fibril forming collagens; these collagens are located in fibrillar structures [24,27]. Note that the fibril-forming collagens in the connective tissues of invertebrates such as sea urchin, as well as sponge, may have more varied structural features than those of the standard fibrillar vertebrate collagens, e.g., triple helical domains of varying lengths [32,33]. Figure 1A presents a sketch of the sea urchin spine-test system containing some known connective tissues; a schematic of the hierarchical architecture of connective tissue is illustrated for the catch apparatus (CA).

Collagenous scaffolds made from ECM-DT have attracted a lot of attention because the scaffold can retain important micro-structural properties [15] and biochemical composition [34] of the native ECM. Besides collagen, the other key ECM components of interest for ensuring that the scaffold can function as intended are glycosaminoglycans and proteoglycans [3]. Since these protein cores of the latter are highly conserved in many species [32,33], their presence in the ECM-DT would help minimize unintended immune response [35]. At the microscopic length scale corresponding to cells, the structural environment is also well-preserved in the ECM-DT; this means that the matrix microenvironment may be effective in directing cellular phenotype via geometric cues [4,36] as well as growth factors for cell attachment, proliferation, migration, and differentiation [3]. Scaffolds made from ECM-DT have been investigated for regeneration in a range of tissues [10,34,37]. These scaffolds have been implemented on heart valve [38], tendon [39] and skeletal muscle [35], to name a few.

As the main components of structural ECM proteins, the fibril forming collagens are able to provide the mechanical support for the body, by an analogy to engineering fibre reinforced composites [26,27,40,41]. These fibrous structures are found in the musculo-skeletal connective tissues, such as tendons [25,27], ligaments [25,26], muscles [42,43], and in skin [41]. The ECM of connective tissues, such as tendons and ligaments, features a hierarchical architecture (Figure 1A) comprising collagen fibres which are bundles of collagen fibrils [27]. The other ECM components, particularly the fibril-associated proteoglycan, such as the small dermatan-sulfate proteoglycans (decorin and biglycan) bound onto collagen fibrils [44], are often thought to facilitate tissue deformation in response to external loads. The main contributor to tissue deformation is the fibril-fibril sliding action [45,46], analogous to the role of compatibilizer in engineering fibre reinforced composites [47]. Both the structure and biochemistry of these proteins are described in considerable detail in other published reports and there is little need to discuss them further here. The reader is directed to the works of Bailey and co-workers [24,48] and others [25,31] for collagen, and Iozzo and co-workers [49] for proteoglycans.

From a biomedical engineering perspective, the key advantage of collagen is that it is normally biocompatible, as with most biopolymers from marine sources [20]. The main concern with synthetic polymers is that they may contain unwanted compounds, especially residue of initiators, that could inhibit cell growth [50,51]. However, the collagens extracted by industrial means from bovine sources such as calf skin and bone could be associated with the bovine spongiform encephalopathy and transmissible spongiform encephalopathy as pointed out in previous reports [52,53,54,55,56]. Alternatively, porous scaffolds composed of jellyfish collagen may be made by freeze-drying and cross-linking with 1-ethyl-(3-3-dimethylaminopropyl) carbodimide hydrochloride/*N*-hydroxysuccinimide; these porous scaffolds also demonstrate higher cell viability than those derived from bovine sources [56]. However, the scaffolds made from the jellyfish collagen reveal similar inflammatory response as those from bovine sources [56]. Di Benedetto and co-workers have developed a method for processing substrate scaffold from native collagen fibrils extracted from the peristomial membrane (a connective tissue) of the sea urchin [57]. The scaffold features a homogeneous fibrous mesh with thickness of around 2 μm [57]; the fibril diameter ranges 30–400 nm [57]. The general architecture of the Di Benedetto scaffold, i.e., fibril organization and bundle orientation, is identical to the structure and organization of collagen observed in several human tissues such as tendon, ligament, cornea, skin and blood vessels [31], as well as other mammalian tissues, such as murine tendons [40,58] and avian tissues, such as chicken tendons [59,60].

While collagenous ECM of the sea urchin possesses structural features and mechanical properties that are similar to those of mammalian ones, even more intriguing is that some tissues such as the ligamentous catch apparatus can exhibit mutability, namely rapid reversible changes in the tissue mechanical properties. These tissues are known as mutable collagenous tissues (MCTs). Although the mechanism of mutability in MCTs is still not clear, progress has been made in the study of the mechanics of fibril sliding in the MCT that could contribute somewhat to our understanding of the mechanical responses underpinning the changes in stiffness in the MCT. In a recent study to assess the non-collageneous content in the interfibriillar matrix, Ribeiro et al. [61] pointed out that the mechanical adaptability of MCT depends on the modulation of interfibrillar cohesion. Barbaglio et al. [62] added that there is good evidence that this is mediated by the GAGs; in the compass depressor ligaments (CDLs), variability in the GAG concentrations is observed (at different pH values) in the respective mechanical states, namely compliant and stiff. This is because in order to withstand unidirectional tensile stresses the CDL has to recruit the appropriate number of interfibrillar linkages (via GAGs) into resisting/facilitating fibril-fibril sliding [62]. Barbaglio et al. [62] concluded that the mechanical adaptability of the MCT may not require (appreciable) changes in the collagen fibrils. To demonstrate this, Mo et al. [63] used high-resolution X-ray probe that measures how collagen fibrils of echinoderm connective tissue stretch, slide, or reorient in real time, to show that the contribution to the changes in the MCT stiffness is dominated by changes in the stiffness of the matrix between individual fibrils, rather than the properties of the fibrils themselves.

In spite of the recent progress on the study of the sliding action of collagen fibrils [62,63], how these findings could point to a nerve-control system for regulating the ECM macromolecules that are involved in the sliding action of collagen fibrils in the MCT [64,65] is still far from clear. The aim of this review is to discuss the findings, from the key early studies to the most recent studies of the basic mechanics of MCT (from a mechanical engineering perspective), addressing the key attributes of the structure and function of the ECM of the sea urchin ligaments that are related to the fibril-fibril sliding action at the respective mechanical states (“standard”, “stiff” and “compliant”)—the focus is on the respective components within the hierarchical architecture of the tissue. Detailed discussion of the system of nervous control, as well as the biochemical composition, that regulates mutability is out of the scope of this review. Thus, the main ECM components of interest here are the collagen fibrils and the interfibrillar matrix components. The discussion will draw findings from experimental studies conducted on sea urchin, the theory of fibre reinforced composites and from the analyses of (non-mutable) connective tissues from other (vertebrate) animals to establish general conclusions concerning the mechanical response of the MCT at specific mechanical states, namely the stiff and complaint states. The overall aim is to enable the development of a de novo understanding of the reinforcement processes in ECM-DT that may result in novel concepts for technological innovation, e.g., in the development of new types of mechanically tunable biomaterials. In the sections that follows, we will address essential concepts concerning the collagenous scaffold design, in the context of ECM, from sea urchin connective tissues. Thereafter we will discuss the biomechanics of collagen fibrils in sea urchin connective tissues in order to illuminate the basis of the structure-function relationship of the ECM of sea urchin connective tissues. Finally, we will conclude the discussion of the sea urchin tissue with reference to a recent framework that has been proposed for addressing the goal of understanding ECM mechanics.

## 2. Collagenous Scaffold Design

### 2.1. Connective Tissues with Properties of Mutability (MCTs)

One of the most intriguing properties of the sea urchin connective tissues, such as the ligamentous CA (Figure 1) [66], is that they can switch from the viscoelastic fluid state to the solid state, reversibly, on a timescale of the order of 1 s [14,15,16,67,68]. Figure 1A illustrates the ligamentous CA and muscles within a spine joint of the sea urchin. Early studies have referred to the different states as “çatch” and “out of catch” [22]. The latest studies have classified these states into three, sometimes renamed as “standard” (normal), “compliant” and “stiff” [69]. The underlying mechanisms regulating these states are often not clearly spelled out. In this review, we present fresh arguments to explain how the stiff state is associated with the elastic stress transfer mechanism (Section 3.3) while the compliant state is associated with the plastic stress transfer mechanism (Section 3.4). As they can change from one state to another in a short span of time, these tissues are regarded as “smart” or “intelligent” tissues [66]. These tissues are also commonly referred to as MCTs to reflect their unusual morphofunctional adaptations [64]. Physically, one finds that these MCTs are responsible for locomotion [70], attachment [70] that includes defining the posture of the animal [67], and even autotomy [71,72]. Interestingly, while autotomy is associated with the compliant state [73,74], the underlying mechanism regulating this is not clear. In this paper, we explore fresh arguments from a molecular perspective and from the mechanics of fibrillar failure to show how autotomy could occur following the compliant state; this discussion is covered in Section 3.5.

For practical reasons, the sea urchin spine can point freely in any direction as permitted by the joint; the spine can also be immobilized to the skeletal test [15,17]. Figure 1B illustrates two possible positions that the spine can adopt. The joint at the spine-test system comprises an outer frustum-like (i.e., ring-like) muscles and an inner frustum-like (i.e., ring-like) CA connective tissue [14,15,17]. The former is responsible for spine movement by synchronized action involving contraction and relaxation; the latter is for controlling the spine orientation by resisting stretch [15,17]. The interplay between the actions of the muscle and CA enables the spine to carry out locomotion, as well as to respond to an external mechanical stimulus in two ways known as the convergence response and the freeze response [14,17,75,76]. When the skeletal test surface is mechanically stimulated, the spines around the stimulated area are provoked to lean down (Figure 1B) to cover that area [75]; when the spine is mechanically stimulated, it becomes immobilized in an upright position [75]. For locomotion/attachment/posture maintenance, in order to move the spine, first, the CA has to become compliant to allow the ring of muscles to position the spine that enables it to brace against marine surfaces such as the rocky wall of a cozy reef crevice. Second, the CA has to stiffen to lock the spine into position and then the muscles relax. By allowing for several spines to stiffen collectively, this makes it difficult to dislodge the sea urchin without breaking the spines and the skeletal test. This behaviour of the spine has raised some interesting questions. For instance, while the ligament can stiffen in any position of the spine (Figure 1B), does this imply that the entire tissue is straight? Additionally, on the side of the spine where the muscle is contracted, the ligament is compressed and therefore shortened (Figure 1B) but no appreciable kinks appear [14]. How does the MCT shorten without kinking? It appears that this has to do with the mechanics of fibril-fibril sliding and the dependence of this mechanism on the length of the collagen fibrils whereby a large proportion of the fibrils could possess lengths that are considerably shorter than the tissue. Details of the basis underlying the fibrillar length-arguments are found in Section 3.2, Section 3.3 and Section 3.4.

The MCT is essentially a complex system consisting of two main components, namely the collagen (in fibrillar forms) which is embedded and organized within an ECM containing nonfibrillar ECM components, such as proteoglycan and glycoprotein [77]. In particular, there are several types of proteoglycans—of interest in this review are those that are believed to bind to the surface of collagen fibrils via core proteins, e.g., small dermatan-sulfate proteoglycans [44,78]. To a large extent, the ultrastructure of the MCT [22,66] bears some resemblance to the connective tissues of humans [40,58]. The simplest explanation for the mutability of the MCT is that the collagen fibrils are able to slide relative to one other, with the help of the proteoglycan [79], to execute tissue length changes (“out of catch” phase) but are inhibited from sliding when the tissue is in “catch” [22]. It then follows that the mechanical states of the tissue are mediated by the interactions of collagen fibrils with the surrounding matrix [69] governed by elastic stress transfer at low loads [80,81] and plastic stress transfer at higher loads [80,81]. (These mechanisms will be discussed in subsequent sections.) The interactions are in turn under the control of a nervous system, regulated by soluble molecules that are secreted locally by neurally controlled effector cells [64,65]. Stiparin, a glycoprotein of ECM, has been reported to result in the aggregation of isolated fibrils and has initially been identified as a tissue-stiffening factor [65]. A second glycoprotein, i.e., an inhibitor of stiparin, was shown to bind to stiparin, to inhibit stiparin’s ability to induce fibril aggregation [82]. More recently, tensilin, a third component, is believed to induce collagen-fibril aggregation in vitro, as well as to stiffen the tissue [66,70,73]. While a detailed molecular mechanism for the regulation of collagen-fibril associations in the sea cucumber dermis has yet to be developed, it also raises the question of whether any related phenomena occur in vertebrate tissues.

The findings concerning the mutability of these tissues have inspired the development of “smart” materials with dynamic properties, as well as having the capacity to control the change in properties in response to environmental demands. With regards to biomedical engineering applications, Trotter and co-workers [23] have proposed to design a new biomaterial made from collagen fibrils embedded in a synthetic matrix material (Section 2.2). The fibrils may be obtained from the sea cucumber dermis [23]. What is the applicability of these smart materials? It has been suggested that dynamic MCT-like materials may be useful for making scaffolds for tissue engineering where the regenerating tissue requires a microenvironment that can dynamically change to match the requirements of the cells [83]. For instance, in regenerative applications of stem cells, such as tissues for postmyocardial infarction patients, the microenvironmental elasticity of the scaffold can direct the native mesenchymal stem cells to specify lineage and commit to phenotypes [4]. Dynamic biomaterials may also find applications in the uterine cervical tissue of mammals—dynamic changes that involve remodelling the tissue to enable the tissue to be become very compliant so as to facilitate the labor process although these changes happen on a longer timescale, i.e., long before the onset of labor [84]. Current MCT-like materials have been produced using shape-memory polymers; these materials attempt to mimic the MCTs’ ability to change mechanical properties “on command” with reversible capability when subjected to a stimulus such as a change in temperature [64]. A new biomimetic research strategy has been proposed to further characterize the properties of MCTs so as to gain deeper insights—the aim is to develop innovative ECM biomaterials with dynamic mechanical properties that finds applications in vitro as well as in vivo [57,85].

### 2.2. Structural and Mechanical Compatibility

**Figure 2:**
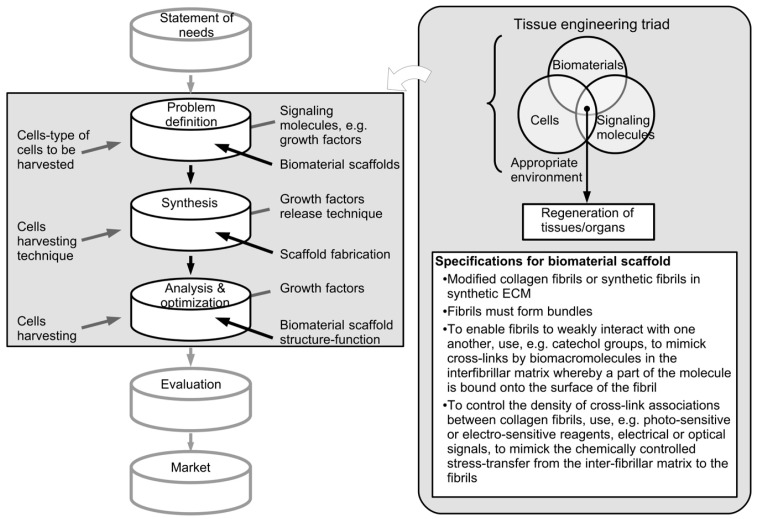
The design process for a tissue engineering approach. **Left panel** shows a flow-chart of the design process. The focus in this process is on the biomaterial for the scaffold development (highlighted in dark fonts). The flow of the design process is typical of engineering design, with the following key stages, statement of needs, problem definition, synthesis, analysis and optimization, evaluation and, finally, market [87]. Of note, some of the stages are expected to be iterative. **Right panel** shows the tissue engineering triad, comprising biomaterials, cells and signaling molecules. The engineering triad is linked to the problem definition stage and continues through to the analysis and optimization stage. The desired specifications for the biomaterial scaffold are outlined in the box based on some of the key arguments developed by Trotter and co-workers [23].

The process of scaffold design for tissue engineering has attracted many studies [86]. From a mechanical engineering perspective, the key design stages that encompass the process of scaffold design address needs recognition, problem definition, synthesis of ideas, analysis and optimization, evaluation of the prototype performance and manufacturability, and finally, bringing the product into the market. These key stages can be encapsulated in a design flow-chart (Figure 2) [87]. The overall direction of the flow in this chart is aimed at achieving a final product that is usable but one may also expect that some stages could progress iteratively, which is typical to any design process.

In the ECM design process (Figure 2), the recognition of needs should address the desire for the scaffold to mimic the ECM of the native tissue. The problem definition stage then identifies the desired mechanical properties, structural features and other biocompatibility issues. The synthesis stage combines the novel ideas, contrived to address the problem definitions, for making the scaffold. The analysis and optimization stage implements the design of experiments; the results from these experiments are used to assess the performance of the scaffold. In the evaluation stage, based on the selected concept and the information about the performance of the ECM materials, processing and costs, the scaffold is evaluated to find out how the proposed design fulfils the specification and if so, further evaluation by clinical trials may be appropriate to confirm the functionality of the design before the product is released to the market.

The relationship between the tissue engineering triad and the design process is indicated in Figure 2. In essence, the tissue engineering triad could span from the problem definition stage to the analysis and optimization stage. Among the three components of the tissue engineering triad, for the purpose of this discussion, we shall only be concerned with the biomaterials component. At the problem definition stage, considerations for the collagen biomaterial would address the respective structural and mechanical properties of the target tissue, by identifying the key responses of the tissue under an external load and the range of values. Underlying the key responses are concepts related to the biomechanics of collagen (Section 3) and non-collagenous components (Section 4.6). At the synthesis stage, this could involve the exploration of different fabrication methods, e.g., reconstituted collagen and ECM material from decellularised tissue, to address the suitability of the respective methods for satisfying the constraints associated with the mechanical considerations. The analysis and optimization stage provides experimental investigations, based on a design of experiment, into the performance of prototypes developed using the methods identified in the synthesis stage. The development of the optimized solution would require careful interpretation of the findings derived from the experimental data. The interpretation process is expected to involve revisiting the premises underlying the arguments for the problems identified for the triad components in the problem definition stage.

What are the desirable ECM components for a ECM-DT? As pointed out in Section 1, Trotter and co-worker have proposed an ECM scaffold with MCT-like properties comprising bundled parallel collagen fibrils that are organized by collagen fibril networks for the biomaterials component at the problem definition stage [23]. The desired specifications are listed in Figure 2. To address both the problem definition stage and the synthesis stage of the design process, Trotter and co-worker recognized that the fibrils have to aggregate to form bundles; in order to enable aggregation these fibrils would have to be weakly interacting with one another [65,82]. In the echinoderms, such as the sea cucumber, stiparin glycoprotein is believed to be responsible for the aggregation of the fibrils in the MCT [23,65]. For a ECM-DT, the strategy is to apply a stiparin-inhibitor that can bind stiparin and inhibits the capacity of stiparin to bind to the fibril [23]. So far the inhibitor component has yet to be conclusively identified. For a synthetic analogue, the challenge is to develop a method that can chemically control the stress transfer from the interfibrillar matrix to the fibrils. For instance, photo-sensitive or electro-sensitive reagents could be introduced to bind covalently to fibril surfaces and form cross-links between adjacent fibrils, in order to cause the ECM to stiffen, as well as to reverse the effects, when required [23]. In any case, the matrix surrounding the fibrils should contain macromolecules that can form cross-links between the fibrils and macromolecules that can have a reversible effect. To implement cross-links for a synthetic ECM scaffold with MCT-like properties, catechol related polymers such as polyacrylamide complexed with phenylboronic acid, have been suggested [23]. It is often argued that to form cross-links, the interfibrillar matrix could contain macromolecules whereby a part of the a molecule is bound onto the surface of the fibril [23]. Hynes and Naba [88] have suggested that parts of glycosaminoglycans and proteoglycans could be bound onto the fibril surface in the MCT, drawing on parallel arguments developed for vertebrate tissues. In the case of the synthetic ECM scaffold with MCT-like properties, the catechol group could be a possible candidate for binding onto both the modified collagen fibrils as well as the synthetic fibrils [23]. In connective tissues, controlling the number of cross-link associations between collagen fibrils is important for regulating interfibrillar shear stress [80,81], underlying the mechanics of the tissue [89,90]. In the sea-urchin, it is found that a nervous input could cause the resident cells to release stiffener macromolecules, which diffuse into the interfibrillar space, and bind to the fibrils to creates strong and numerous interactions between them [69,73,74]. For engineering a synthetic MCT-like scaffold—if photo-sensitive or electro-sensitive reagents are to be used—electrical or optical signals may be applied as inputs to change the redox potential of the matrix to affect the binding between the catechol and boronic acid moieties. Finally, a strategy is required to bind the fibres into bundles by a matrix which can result in a scaffold that may then be modelled by elastomeric properties. In the MCT, this matrix is observed to be predominantly fibrillin microfibrils [23,66,69,70,82,83,91]. However, for the synthetic MCT-like scaffold, an elastomeric hydrogel strategy may be needed to act as a substitute for the fibrillin microfibrils [23].

There are more than twenty different classes of techniques for fabricating tissue-engineered scaffolds [1]. Some of these techniques are known as solution casting, gel-pressing, microspheres, macro-porous beads, particle-aggregated scaffolds, freeze-drying, acellularization, electrospinning, and wet spinning; some of these may be used in combination for producing an ECM-like scaffold. Thus, one may attempt to extract collagen by mixing neonatal human dermal fibroblasts with acid-soluble type I collagen. The extracted collagen is then cast into a desired shape in a (Teflon) mold, followed by culturing for 4 days to allow compaction—which also stiffens and aligns the gel. This method can result in a collagen gel containing a heterogeneous population of fiber diameters (42 to 255 nm, mean diameter ~69 nm) [92]. Alternatively, electrospinning [93] can be employed to produce fibrous scaffolds comprising type I collagen fibers—with diameter comparable to native collagen fibrils—which also exhibits the 67 nm banding pattern that is characteristic of native collagen [94]. Additionally, the pressurized gyration [95,96], a method that involves rotating a perforated pot containing a reservior of polymer solution at high speed, as well as the pressurized melt gyration [97], a powerful method that can lead to high production rate, ease of production, and highly controlled fiber morphology, may also be considered for producing collagen fibrous scaffolds.

How then are ECM-DT scaffolds produced? As pointed out in Section 1, ECM scaffolds can be obtained from allogeneic or xenogeneic (segmented or whole tissues) sources by treating the isolated tissue with decellularizing solution and incubating for cell lysis [1]. The processed scaffold may be regarded as an “entry-level” ECM-DT, i.e., a ECM-DT that is possibly devoid of some key components such as stiparin-inhibitor and catechol related polymers that are essential for regulating the fibrillar structural organisation. Based on the methods adopted by Yang et al. [12] and Kayed et al. [11] for treating a given thin tissue, or the method adopted by Di Benedetto et al. [57] for treating a given minced tissue, the specimen is washed for about a day in a hypertonic buffer (PBS with Triton X100 (TX) and EDTA solution, or Tris with EDTA). Rinsing may take place in a low temperature environment; the hypertonic solution is also continuously agitation. Thereafter the sample is rinsed in a decellularising buffer (PBS or Tris with SDS). For a minced tissue specimen, this solution is then replaced by a disaggregating solution (one containing e.g., Tris and EDTA); a collagen suspension is obtained which is then filtered and purified using a EDTA solution, followed by distilled water. For long-term storage of the suspension, one could dry them in silicone molds. In the case of the thin tissue, after treating in the decellularising buffer, the specimen is stored away in PBS until it needed.

## 3. Collagen Fibril Biomechanics

### 3.1. Stress-Strain Relationship of MCT

**Figure 3:**
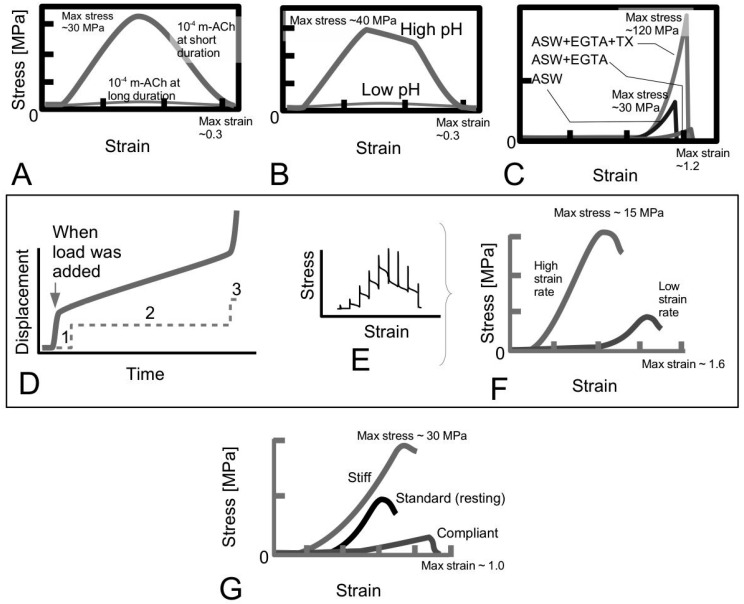
Profiles of the stress versus strain curves of mutable collagenous tissues (MCTs). (**A**) A sketch of the graph of stress versus strain of the CA, sea urchin (*Anthocidaris crassispina*) [15]; (**B**) A sketch of the graph of stress versus strain of the catch apparatus, sea urchin (*Anthocidaris crassispina*) [16]; (**C**) A sketch of the graph of stress versus strain of the tube feet tissue, sea urchin (*Paracentrotus lividus*) [70]; Sketches of (**D**) the graph of displacement versus time, indicating the primary (#1), secondary (#2) and tertiary (#3) phases; thereafter rupture results; (**E**) the graph of incremental stress versus strain and (**F**) the graph of stress versus strain (derived from **E**) of the compass depressor ligament, sea urchin (*Paracentrotus lividus*) [66]; (**G**) Sketch of graph of stress versus strain of the dermis of the sea cucumber (*Cucumbria frondosa*) [23] for the purpose of comparison with the results from the sea urchin (**A**–**F**). Symbols in the graphs: ACh represents acetylcholine; ASW, artificial sea water; EGTA, ethylene-*bis*-(oxyethylenenitrilo)-tetraacetic acid (calcium chelator); TX, Triton X100.

The response of the MCT to an external load has been a subject of many studies. In this section, we examine three different tissues from the sea urchin, highlighting the mechanical properties of these tissues in the context of their stress-strain behaviour. These tissues are the CA, compass depressor and tube feet. To begin, Hidaka and co-workers [15,16] have evaluated the mechanical properties of the CA tissue of the sea urchin (*Anthocidaris crassispina*) to varying physico-chemical factors (pH, neurotransmitter) in order to gain insights into the underlying mechanisms regulating the ability of the CA to execute mutability. In one study, at a displacement rate of 7 μm/s where the viscous resistance is expected to predominate in the CA, it was found that the pH and neurotransmitter acetylcholine (ACh), has a significant effect on the viscous resistance. Figure 3A,B show the stress-strain curves of the CA obtained at varying ACh and pH values, respectively. In particular, high pH value results in high stress uptake in the CA [16]. The duration of ACh-treatment is shown to affect the stress uptake in the CA—the longer the treatment the lower the stress uptake [15]. In all cases, the stress-strain curve shows a non-linear increase in stress with strain from initial loading (the toe region), followed by a somewhat linear increase in stress with strain, a point of yielding and thereafter the point of maximum stress; beyond this point, failure in the tissue results in a somewhat gradual decrease in stress with increasing strain. The highest stiffness observed in these results is estimated at 200 MPa while the highest maximum stress is at around 30 MPa. It is important to note that the profile of the stress-strain curve resembles those from mammalian connective tissues such as mouse tail tendons [40,58] and sheep anterior cruciate ligaments [26], where the tissues have also been subjected to loading along their axes at displacement rates of order of 10 μm/s.

Investigation on the mutability of MCT by examining the effects of ions in the ECM have been reported. Figure 3C shows stress-strain curves of the tube feet tissue of the sea urchin (*Paracentrotus lividus*) treated in the following respective bathing solutions, namely artificial seawater (ASW, regarded as a standard solution), ethylene-*bis*-(oxyethylenenitrilo)-tetraacetic acid (EGTA), and TX with EGTA, to identify the properties related to the mutability of the tube feet tissue [70]. Of note, EGTA acts as a calcium chelator to remove the endogenous calcium from the tissues; TX is a non-ionic detergent that can disrupt cells in the tube feet. When the tissues were tested to rupture at 25 mm/min, all tissues exhibit J-shaped profiles. In particular, the J-shaped profile begins with a long low-stress toe region, followed by a rapidly increasing stiffness with increase in strain up to the point of maximum stress and, thereafter, a rapid decrease in stress. (Of note, the displacement rate of 25 mm/min corresponds to 417 μm/s, which is 400 times higher than those used in the investigation for Figure 3A,B) When the tissue is treated to calcium removal solution (ASW + EGTA), the tube feet tissue results in a dramatic decrease in strength, stiffness and toughness with respect to the control (i.e., in standard solution only). On the other hand, treatment with ASW + EGTA + TX solutions reveals a dramatic increase in strength, stiffness and toughness with respect to the control. The highest stiffness observed in all these results is estimated at 400 MPa; the highest maximum stress occurs at around 120 MPa. These findings suggest that the mechanical properties of the tube feet tissue are affected by the calcium ions and the juxtaligamental-like cells in the tissue. For instance, the tissue becomes compliant in the absence of calcium. The calcium-removal test suggests that ions in the ECM of the MCT play an important role in regulating the tissue mechanical properties and warrants further discussion; this is addressed in Section 4.6 with regards to the effects of the composition of the interfibrillar matrix on fibril-fibril interactions.

To examine the viscoelastic behaviour of the MCT, Figure 3D shows a sketch of the graph of the displacement versus time to illustrate the creep response of the compass depressor tissue of the sea urchin (*Paracentrotus lividus*) [66]. Evaluating the second phase of the creep response curve, the mean coefficient of viscosity is found to be 561 ± 365 MPa·s [66]. The massively large standard deviation reflects the large variability in the values coefficient of viscosity (104 to 1477 MPa·s) derived from this study for a sample size of 31 [66]. Figure 3E,F show the sketches of the graphs of stress versus strain of the sea urchin compass depressor tissue [66]. The stress-strain curve (Figure 3F) has been derived from the incremental stress-strain approach (Figure 3E). To this end, each point on the graph of Figure 3F represents the peak stress value of the incremental curve (Figure 3E); all curves results in the toe, linear and yield regions similar to those of Figure 3A–C. Assuming the length of the compass depressor is of order of 10 mm based on the images shown in the report [66], these curves correspond to displacement rates ranging 0.07 to 0.6 mm/s. The highest stiffness estimated from these results is 20 MPa; the highest maximum stress occurs at around 15 MPa. The profiles of the respective curves are also comparable to those of mammalian tissues, such as skin, that have been investigated for viscoelastic effects [41].

Figure 3G shows a sketch of the graph of the stress versus strain of the dermis from the sea cucumber derived at the respective state, namely stiff, standard (i.e., intermediate/resting) and compliant states [23]. These sketches are shown here for the purpose of comparison with the stress-strain curves from the three different tissues of the sea urchin. As expected, the stress versus strain curve shows a very steep gradient for the tissue in a stiff state as compared to the tissue in a compliant state; the peak stress is also higher in the former than in the latter tissue. Of note, Trotter and co-worker have referred to the standard and compliant states as the intermediate and plastic states, respectively [23].

Table 1 tabulates the findings of the mechanical properties of the tissue of the sea urchin that have been discussed in the previous paragraph. Thus, among the three different tissues of the sea urchin, the compass depressor tissue exhibits the smallest strength (maximum stress) and stiffness while the tube feet tissue exhibits the largest strength and stiffness. The largest and smallest extensibility (maximum strain) correspond to the compass depressor and CA tissues, respectively. Of course, the varying magnitudes of the respective mechanical properties of different tissue highlighted here may be attributed to the different organisation of the network (orientation and packing) of the collagen fibrils as well as the structural properties (e.g., aspect ratios) of the fibrils. Nevertheless, these values are of similar order of magnitudes to those of mammalian tissues [27].

### 3.2. Shear Action Underpins the Mechanism of Collagen Fibril Reinforcement of MCT

In this section, we discuss how shear of the collagen fibrils enables the fibrils to take up stress and contribute to resisting the external load acting on the MCT that tends to pull it apart.

The fundamentals of fibre-reinforced composites have been well-established for engineering composite materials, leading to analytical solutions where linear elastic properties of the materials for the fibre and matrix are concerned [98]. However, attempts to apply these concepts to collagen fibrils in biological tissues are challenged by uncertainties surrounding the nature of the ECM components, particularly the degree of non-linear properties of these components [99]. Here we shall adapt the key features of the basic models [80,81,100] that have been established for explaining how collagen fibrils provide reinforcement to ECM of connective tissues to describe the ECM of MCT.

**Figure 4:**
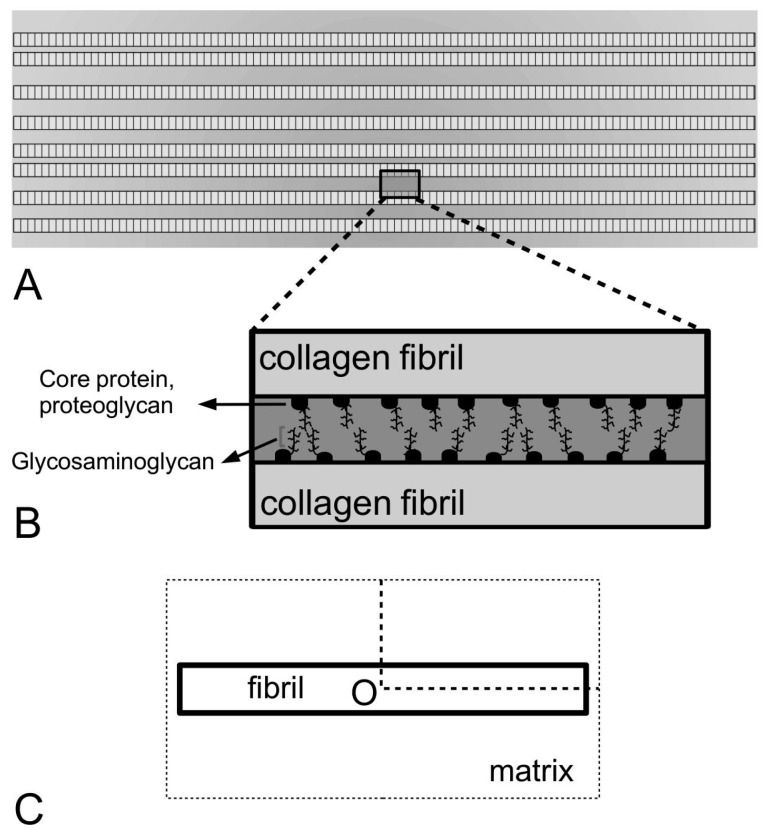
General model of collagen fibril in extracellular matrix (ECM). (**A**) An array of parallel collagen fibrils embedded in the ECM. The vertical dard bands and light shades represent the D-periodic patterns. (**B**) Interaction of collagen fibrils in the matrix. Here the interaction is assumed to be aided somewhat by proteoglycans and glycosaminoglycans, although the exact identity of the proteoglycans has yet to be determined. Not shown in this schematic are the glycoproteins. (**C**) A single collagen fibril modelled as a uniform cylinder. The fibril centre, O, defines the origin of the cylindrical polar coordinate system (*r*,θ,*z*), where the *z* axis coincides with the axis of the fibril. Of note, the single fibril-matrix model in part C provides the basic “template” for many of the discussions in this review where stress uptake in the fibril is the key concerned (see Figures 7A and 10A for similar schematics).

As shown in Figure 4A, consider an array of collagen fibrils, parallel to one another, embedded in the interfibrillar matrix of the MCT. In these models, the fibrils will always be considered to be arranged in the direction of the tissue axis. The applied force on the tissue will always be considered to be acting along the direction of the tissue axis. At closer view, between any two fibrils are proteoglycans/glycoproteins associated with the fibrils (Figure 4B); these protein macromolecules are assumed to be involved in regulating the transfer of stress from the matrix to the fibril as well as between the fibrils. It is also assumed that (1) there are numerous such proteoglycans/glycoproteins; (2) bonds (e.g., van Der Waals, hydrogen) exist between proteoglycans/glycoproteins on adjacent fibrils; (3) these proteoglycans/glycoproteins are distributed uniformly over the fibril/matrix interface so that continuum mechanics can be used to analyze the problem [80,81,100]. For simplicity, most models are based on solving the stress in a single collagen fibril embedded in the matrix as illustrated in Figure 4C. Suppose the fibril is parallel to the axis of the tissue; additionally, an external tensile load acts along the axis of the tissue. At initial loading, as the interfibrillar matrix deforms in shear, this generates shear stresses on the surface of the collagen fibril [80,81,100]. Interactions of ECM components in the interfibrillar matrix (i.e., proteoglycans/glycoproteins) with those associated with the collagen fibril then cause the fibril to deform axially [80,81,100].

**Figure 5:**
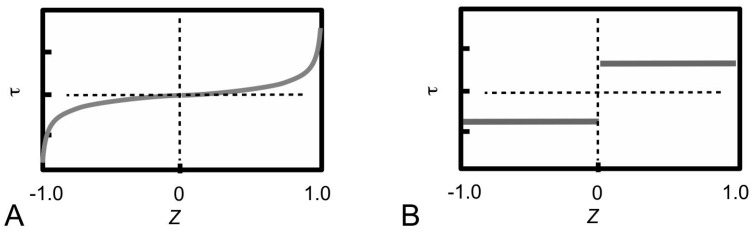
Fibre-matrix interfacial shear stress, τ, distributions [99,101]. (**A**) Shear-lag model; (**B**) Shear-sliding model. Here *Z* represents the normalized coordinate, i.e., *Z* = *z*/*L*_CF_, where *z* is the *z* coordinate of the cylindrical polar coordinate system and *L*_CF_ represents the half-length of the fibril. *Z* is used to describe the distance along the fibre axis from the fibre centre, *Z* = 0, to the respective fibre ends, *Z* = 1 or −1.

From several studies in the early 1990s, it was clear that different mechanisms of stress transfer in the ECM result in different stress uptake in the fibre [99,101]. According to Aspden [99,101], the manner in which an elastic fibre takes up stress depends on the interfacial shear stress (τ) distribution. This results in two different approaches to solving the stress in the fibre. The first approach, known as the shear-lag approach [98,102], is associated with the τ distribution illustrated in Figure 5A; the second approach, known as the shear-sliding approach [103], is associated with the the τ distribution illustrated in Figure 5B.

In the shear-lag approach, the τ is a minimum at the fibre centre (*Z* = 0; Figure 5A) but increases non-linearly from the centre to a maximum value at the respective ends (*Z* = 1 or −1; Figure 5A) [104,105,106]. In the shear-sliding model, τ is constant throughout the fibre surface (Figure 5B) [105,107,108]. These descriptions are not based on a detailed analysis of the viscoelastic behaviour of the tissue, but the simplicity of these concepts has facilitated general conclusions to be drawn concerning the reinforcement of the MCT by collagen fibrils. In particular, both shear-driven arguments have been applied to evaluate the stress uptake in collagen fibrils [27,80,81]. More recently, Szczesny and co-workers have sougth to further establish the role of the interfibrillar matrix in ECM that can yield deeper insights into the elastic and plastic stress transfer mechanisms [89,90,109]. In particular, Szczesny and co-workers carried out a series of experiments to study the viscoelastic behaviour of tendon fascicles by evaluating the contribution of the ratio of the strains of a fibril to the whole tissue during stretching using an incremental relaxation approach [89,90,109]. In the following sections, the basic concepts underpinning the elastic and plastic mechanisms of stress transfer are elaborated.

### 3.3. Interfibrillar Shear Response by Elastic Stress Transfer Directs the Stiffening of the MCT

The purpose of this section is to present the key arguments to highlight how elastic stress transfer mechanism directs the stiffening of the MCT.

Based on the general conclusions drawn from the study of soft connective tissue reinforced by collagen fibrils, when a load is applied to the MCT in the passsive mode where mutability is absence, the tissue is likely to encounter elastic stress transfer process during initial loading when the load is low [38,39,88]. In other words, the interfibrillar matrix and collagen fibril respond elastically to the external load; they are able to return to their original structural state when the load is removed [38,39,88]. The difference in the elastic moduli of the two components plays an important role in influencing the differential axial elastic displacements in the fibril and in the interfibrillar matrix [38,39]. Consequently, shear strains are produced on all planes parallel to the axis of the fibrils in the direction of this axis [38,39]. The above description of the state of the individual ECM components during elastic stress transfer may then be applied to the situation where mutability is considered, when the MCT exhibits the stiff state following a transition from the compliant state. Thus, it is hypothesized that elastic stress transfer directs the MCT to take up load to maintain the stiff state.

**Figure 6:**
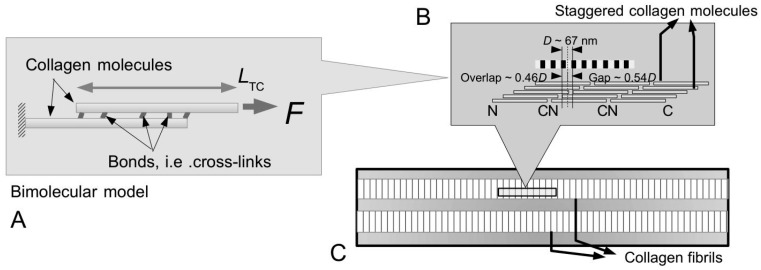
Schematic of collagen molecules in tension in collagen fibrils. (**A**) the Buehler bimolecular model [110], i.e., two collagen molecules sliding under a tensile force, *F*. Symbol *L*_TC_ represents the length of the molecule; (**B**) the axial-staggering of collagen molecules in a fibril. The staggered arrangement gives rise to light-dark bands (i.e., the D-periodic patterns) along the collagen fibril. Symbols *D* represents the *D* period of the collagen fibril; N and C denote the amino-terminus (containing an amine group) and C-terminus (containing carboxyl group) of the collagen molecule, respectively; (**C**) Two adjacent collagen fibrils.

How does collagen provide reinforcement to the MCT in the stiffened state? At the molecular level, consider two adjacent collagen molecules located within a collagen fibril (Figure 6A). Suppose the MCT as a whole is subject to a strain of ε in the direction of the fibril. Two modes of deformation have been predicted using the bi-molecular mechanics approach; the first mode is known as the homogeneous shear while the second is known as the nucleation of slip pulses. The second mode will be elaborated in Section 3.5 when we consider how the fibril could fracture. The homogeneous shear mode explains how the collagen molecules undergo sliding motion when a tensile load acts on the collagen fibril (Figure 6A). Let τ_TC_ represents the shear resistance between the two molecules and *L*_CL_ the contact length between two adjacent molecules. Let *F* be the axial force generated within the molecule, which parameterizes the resistance to the shear action. To order of magnitude, we can identify the *F* with the product of τ_TC_ and *L*_CL_, i.e.,

*F* = τ_TC_*L*_CL_, (1)

or otherwise,

*F* = ητ_TC_*L*_TC_, (2)

where *L*_TC_ is the length of a collagen molecule and η = *L*_CL_/*L*_TC_. The stress, σ_TC_, associated with *F* is,

σ_TC_ = *F/*α_TC_, (3)

where α_TC_ is the molecular cross-sectional area. The homogeneous shear mode assumes that the shear deformation is uniformly distributed throughout the interface of any two collagen molecules; this is expected to occur during initial loading. By virtue of the axial staggering of the collagen molecules, *F* increases linearly with *L*_CL_. Upon evaluating a multiscale model numerically—where the lower length scale level addresses the contribution of the interactions of atoms in the respective molecules to molecular deformation, and the next higher length scale level addresses the contribution of the sliding action of the molecules to the fibril deformation—Buehler has shown that the stress developed in the fibril follows a linear response to increasing strain up to a strain of 0.05 [110,111]. Interestingly, there is no appreciable toe region at initial loading [110,111].

At the fibrillar level, how collagen provides reinforcement to the MCT in the stiffened state may be explained by the stress in the fibril. The first step here is to consider the MCT as a whole, subjected to a strain of ε in the direction of the axis of the tissue. Let *L*_CF_ be the half-length of a collagen fibril and *Z* (=*z*/*L*_CF_) be the normalized axial coordinate (*z*) which parameterizes the axial distance of the fibril starting from the fibril centre (*z* = 0) and ending at the fibril end, i.e., *z* = *L*_CF_. It follows that the rate of change of the axial stress (σ*_z_*) at any point along the fibril is proportional to the difference between the axial displacement of the fibril (*u*_CF_) at that point within the fibril and the corresponding axial displacement of the interfibrillar matrix at the same point if the fibril were not presence, (*u_m_*) [80],

dσ*_z_*(*Z*)/d*Z* = *H*[*u*_CF_ − *u_m_*], (4)

where *H* is constant. For simplicity, as well as for the purpose of this discussion, we shall present the results for uniform cylindrical fibrils. Let *A*_CF_ be the cross-sectional area of the (i.e., uniform cylindrical) fibril, *r*_0_ the fibril radius, and *r_m_* the average radius of the interfibrillar matrix surrounding the fibril. Solving Equation (4) for uniform cylindrical fibrils, one finds that σ*_z_* and interfacial shear (τ) stress generated at the collagen fibrillar surface are given by [27,80]

σ*_z_*(*Z*) = *E*_CF_ε[1 + cosh(β_Cox_{*Z*})/cosh(β_Cox_)], (5)

**Figure 7:**
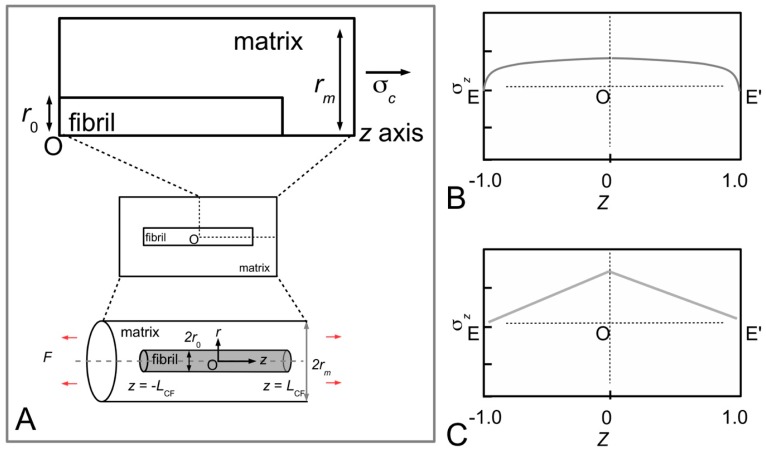
Collagen fibril axial stress, σ*_z_*, distributions. (**A**) Model of connective tissue featuring a collagen fibril embedded in ECM. The proposed interfacial shear stress distributions in the (**B**) Shear-lag and (**C**) Shear-sliding models for collagen fibril biomechanics [99,101]. In part B and C, symbols *F* represents the force acting on the ECM (red arrow represents the direction of F); σ*_c_* represents the stress acting on the tissue in the direction of the fibril, *r_m_* represents the radius of the matrix surrounding the fibril; *r*_0_ represents the radius of the fibril; *L*_CF_ represents the half-length of the fibril; *r* and *z* are coordinates of the cylindrical polar coordinate system; *Z* represents the normalized coordinate of *z* (i.e., *Z* = *z*/*L*_CF)_ which is intended to describe the fractional distance along the fibril axis from the fibre centre, *Z* = 0 (i.e., O), to the respective fibre ends, *Z* = 1 or −1; E and E’ represent the ends of a fibril (Figure 6).

τ(*Z*) = *E*_CF_ε√(*G_m_*/[2*E*_CF_ln(*r_m_*/*r*_0_)])sinh(β_Cox_[*Z*])/cosh(β_Cox_), (6)

where,

β_Cox_ = √(*G_m_*2π*L*_CF_^2^/[*A*_CF_*E*_CF_ln(*r_m_*/*r*_0_)]), (7)

which is written in terms of the collagen tensile stiffness, *E*_CF_, the shear modulus of the interfibrillar matrix, *G_m_*, and the average strain in the fibril, ε. Figure 7B illustrates a typical axial stress distribution in the fibril under tension during elastic stress transfer. This profile applies to a fibril which possesses a uniform cylindrical shape, which is a common assumption for many tissues where the ends could not be observed. Thus, the shear-lag model predicts that the stress in the collagen fibril peaks at the fibril centre and decreases non-linearly to zero at the fibril end. In particular, the decrease is (1) gradual for a large portion of the fibril, around the fibril centre, (2) rapid towards the fibril end. For fibrils which possess tapering ends, the stress distribution profile has been predicted to differ appreciably from those of uniform cylinder [80,81]. This will be discussed further in Section 4.2.

Thus, Equations (3), (5) and (6) provide the basis for fibril reinforcement of MCT during elastic stress transfer. In the stiff state, sliding among the fibrils is greatly reduced [14,15]—the stiffness of the MCT is of the order of hundreds of MPa. In order for this to be maintained, the magnitude of τ must be high. According to Equation (6), a high shear modulus (*G_m_*) would be needed to enforce this.

### 3.4. Interfibrillar Shear During Plastic Stress Transfer Directs the Compliance of MCT

The purpose of this section is to present the key arguments to highlight how plastic stress transfer mechanism directs the compliance of the MCT.

According to the general conclusions derived from the mechanics of soft connective tissue reinforced by collagen fibrils, when an external load acts on the MCT in the passive mode (where mutability is subdued), plastic stress transfer predominates at a sufficiently high load level [27,80,112]. During plastic stress transfer, the interfibrillar matrix around the fibril becomes plastic, bonds at the fibril-matrix interface are disrupted and the interfibrillar matrix “shear-slides” over the surface of the fibril [27,80,112]. Where mutability is involved in the mechanical response of the MCT, Section 3.3 highlights the arguments for how the case of a stiffened MCT is directed by elastic stress transfer mechanism. If the MCT is in the stiff state, and the situation dictates a transition to a compliant state, the description of the state of the individual ECM components during plastic stress transfer may be applicable to the MCT in a compliant state. Thus, it is hypothesized that plastic stress transfer directs the MCT to maintain the mechanical integrity when it is in a compliant state.

How then does collagen provide reinforcement to the MCT in the compliant state? At the fibrillar level, the stress uptake in the fibril undergoing plastic stress transfer may be estimated as follows. For simplicity, the rate of change of the axial force associated with σ*_z_* along the fibril is proportional to product of the fibril radius (*r*), *L*_CF_ and the fibril-matrix interfacial shear stress (τ) [97],

d[σ*_z_*(*Z*)*r*^2^]/d*Z* = −2τ*r*_0_*L*_CF_. (8)

It follows that the stress in the fibril (for given shape of the fibril), may be determined by solving the Equation (8). The fibril aspect ratio is defined as

*q* = *L*_CF_/*r*_0_. (9)

Consider the case of a fibril with uniform cylindrical shape (i.e., constant *r* = *r*_0_). Equation (8) is reduced to

dσ*_z_*(*Z*)/d*Z* = −2τ*q*. (10)

Solving Equation (10) using the boundary condition that states that at *Z* = 1, σ*_z_* = 0 (i.e., a stress-free fibre end), the axial stress is given by

σ*_z_*(*Z*) = 2τ*q*[1-*Z*]. (11)

Figure 7C illustrates the axial stress distribution in the fibril under tension during plastic stress transfer. (The graphs also show the variation in the axial stress distribution with respect to four different fibril shapes but for the purpose of the discussion here we shall consider the case of the fibril with a uniform cylindrical shape.) Thus, during plastic stress transfer, the stress in the collagen fibril peaks at the fibril centre and decreases linearly to zero at the fibril end. The peak stress at the fibril centre could increase with increasing load on the tissue [100,113]. When the peak stress reaches the yield strength of the collagen fibril, the fibril could yield. For fibrils which possess tapering ends, the stress distribution profile differs appreciably from those of the uniform cylinder [80,81]. This will be discussed further in Section 4.2.

In the compliant state, the stiffness is reduced by about an order of magnitude, i.e., of the order of around tenths of MPa. Of note, the sliding mechanism prevails in the connective tissues of human and other animals [114]—sliding of collagen fibre bundles, i.e., fascicles, has been observed [115,116] during tissue deformation. For a MCT to change from a compliant to a stiffened state and vice versa, other mechanisms would be involved in regulating this transition process. As pointed out in Section 2.2, one mechanism could involve nerve-control to cause the stiffening/de-stiffening gycoproteins to be released into the ECM [64]. It is postulated that the de-stiffening effects result in mode β, which is characterized by the initiation of interfacial debonding [27]. Debonding starts at the fibril end and propagates along the interface. In addition, as the deforming interfibrillar matrix slides over the fibril surface, this enables frictional stress transfer to take place. Of note, nerve-controlled transition from a stiffened state to a compliant state may be realized more effectively by ensuring that the interfibillar matrix be completely plastic. Thus, mode α, which is characterized by a plastically deforming interfibrillar matrix [27], could also occur. Consequently, this brings the tissue into plastic stress transfer.

It may be argued that in the normal circumstances, mode α, mode β and plastic stress transfer would be required for enabling the tissue to perform the required physiological activity. However, the plastic stress transfer mechanism could result in failure in the tissue. For instance, when predators seek to pry the stiffened sea urchin off from nooks, the tissue could fail as it undergoes loading from elastic stress transfer to plastic stress transfer (Section 3.4), when the external load on the tissue increases to a critical level. In the interim, the tissue could undergo additional intermediate mode of failure corresponding to mode γ [27]. Here, mode γ refers to the initiation of rupture at the debonded fibril end and the propagation of the crack into the interfibrillar matrix rather than along the fibril-matrix interface [27]. These intermediate modes of failure are analogous to those found in engineering composites [105].

### 3.5. Nucleation of Slip Pulse Predicts Collagen Fracture and Tissue Autotomy

The transition from a stiffened state to a compliant state and vice versa is a physiological process that is not expected to induce failure in the MCT. A change in the states of the respective interfibrillar matrix and the fibril-matrix interface is to be expected in the process of undergoing a transition from the stiff to the compliant state (and vice versa). As pointed out in Section 2.2, “stiffener” proteins, namely tensilin [66,70,73] and stiparin [23,64,69] are believed to play a role in regulating the bonding processes. On the other hand, the regulation of the debonding processes is attributed to inhibitors of these proteins [23,73,74]. The respective proteins secreted into the ECM are associated with stiffener/de-stiffener cells present in the MCT [69], controlled possibly by a nervous input [69,73,74]. In particular, to assume a compliant state, tensilin-inhibitors and stiparin-inhibitors would have to act to cause the interfibrillar matrix to turn plastic (mode α) and the bonds at the fibril-matrix interface to be disrupted (mode β) (Section 3.4). Whether the tensilin-inhibitors and stiparin-inhibitors could also initiate the transitional failure mode γ is not clear but it was suggested in Section 3.4 that this could result in rupture across the ECM.

**Figure 8:**
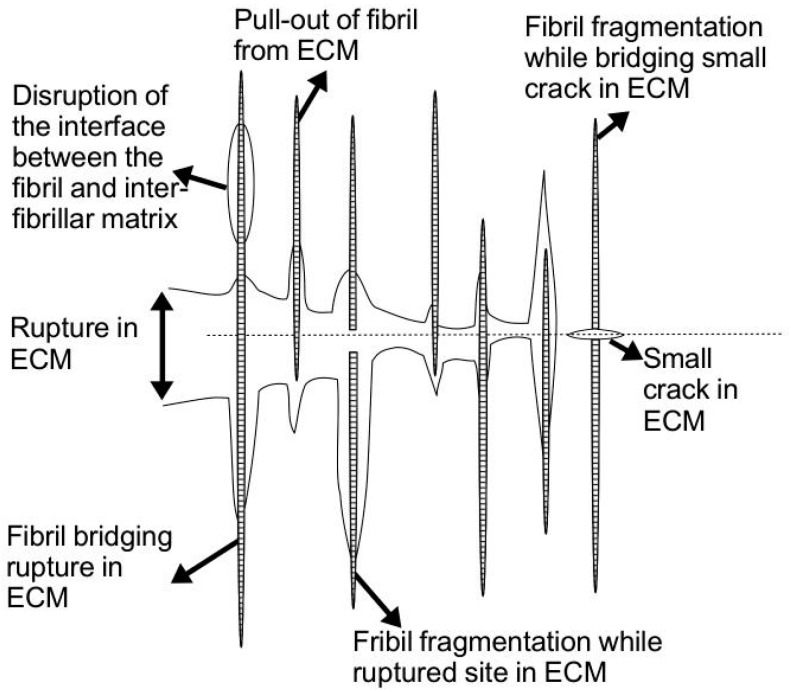
Schematic of tissue rupture. The diagram shows a snapshot of the microenvironment of ECM undergoing failure. These failures are identified as a small crack in ECM, rupture of ECM and bridging of the ruptured site by intact collagen fibrils; at the ruptured site of ECM, fibrils may also be pulled out or fractured. Adapted from Goh et al. [58].

However, if the MCT (in either stiffened or compliant state) is acted upon by an increasing external load (e.g., this may be the result of a predator attempting to pry the sea urchin off from a nook), eventually the tissue fractures (Figure 8) when the stress generated in the tissue, in response to resisting the load that tends to pull it apart, exceeds its fracture strength. Additionally, the spectacular body softening observed in echinoderms following autotomy [68,74] suggests that at the tissue level, the MCT has to break apart following plastic stress transfer. What mechanisms are involved in regulating the fracture of the MCT?

According to the general conclusions drawn from the mechanics of soft connective tissue reinforced by collagen fibrils, in the run-up to MCT fracture, several modes of failures may happen. Namely, fibrils around the matrix ruptured site may experience fibril pullout or fibril rupture (Figure 8) [27,58]. If the fibrils fracture (Section 4.3), the shorter segments that result may continue to take up stress; if the length of these segments are sufficiently long, fracture could still occur when the fracture stress is reached [27,100]. Eventually the fragmentation process terminates because the subsequent fragments generated would not be long enough to take up stress to the level of its fracture stress; the stress transferred to the fibril fragment is insufficient to cause further fragmentation [27,100].

With regards to fibril fracture, the nucleation of slip pulses at the molecular level plays an important role in the dissociation between collagen molecules [100]. The process of the nucleation of slip pulses explains how the rupture of intermolecular bonds, i.e., cross-links in between two collagen molecules (Figure 6A), result in the propagation of slip pulses. For simplicity these cross-links are assumed to be regularly spaced apart [110]. According to Griffith’s fracture energy argument, at the onset of fracture, the criterion for nucleation of slip pulses is dictated by the stress generated by the collagen molecule, σ_TC_, and is of order of the applied tensile stress, σ_Grif_, to cause the MCT to rupture. Let *A*_TC_ be the cross-sectional area of the collagen molecule. Thus, σ_Grif_ is expressed as

σ_Grif_ = √(2*E*_TC_γ_TC_), (12)

where *E*_TC_ is the Young modulus of an individual collagen molecule and γ_TC_ parameterizes the energy required to nucleate a slip pulse [110]. When σ_TC_ < σ_Grif_, the deformation of the collagen molecules is regulated by homogeneous shear (the homogeneous shear theory) between the molecules (Section 3.3). When σ_TC_ > σ_Grif_, nucleation of slip pulses can occur (i.e., the slip pulse theory). Thereafter, a critical molecular length, i.e.,

χ_S_ = [√(2*E*_TC_γ_TC_)]*A*_TC_/{ητ_TC_}, (13)

may be used to determine which of the two cases predominates. Simply, homogeneous (intermolecular) shear predominates if *L*_TC_ ≤ χ_S_; slip pulses predominates if *L*_TC_ > χ_S_. If the tensile force, *F*, in each collagen molecule (Equation (1)) reaches the breaking force of the molecule, *F*_max_, before homogeneous shear could occur or even before slip pulses are nucleated, further occurrence of failure is governed by a second critical molecular length scale,

χ_R_ = *F*_max_/[ητ_TC_], (14)

which determines if the transition from molecular shear-sliding to rupture of collagen molecule can occur. The rupture of collagen molecule predominates if *L*_TC_ > χ_R_; homogeneous (intermolecular) shear predominates if *L*_TC_ ≤ χ_R_. By combining these length scale arguments, it is further proposed that the interplay of the critical length-scales, expressed in the form of a ratio χ_S_/χ_R_, regulate the deformation mechanisms. It follows that slip pulse nucleation predominates at large molecular lengths only when χ_S_/χ_R_ < 1; rupture of the collagen molecule predominates when χ_S_/χ_R_ > 1.

Additionally, the mechanical (as well as chemical) stability of the collagen fibrils may be attributed to the extensive covalent crosslinks [24,48], such as those illustrated in Figure 6A. In general the collagen in the MCT of echinoderm contain higher levels of reducible crosslinks than those found in mammalian collagens [24,48]. However, the levels of the stable hydroxypyridinium crosslinks in the collagen of the MCT are similar to those seen in mammalian fibrous tissues [24,48].

At the length scale of collagen fibril, predictions from in silico models of Buehler [110] and others [111,117] show that beyond a strain of 0.05 (Section 3.3), the stress in the fibril responds non-linearly with increasing strain, reaching a peak stress eventually, and decreasing to zero somewhat gradually as the fibril fractures. Interestingly, the Buehler model [110] predicts that the profile of the stress-strain curves of collagen fibrils is similar to those depicted in Figure 3A,B,F. Altogether these in silico studies suggest that the collagen fibril has a fracture strength of the order of 10^9^ Pa and stiffness of the collagen fibril ranges 6 GPa [110,117,118] to 40 GPa [118].

## 4. Structure-Function Relationship

### 4.1. Vertebrates and Invertebrates with Spindle-Like Collagen Fibrils

As observed in transverse electron micrographs, the cross sections of the MCT, at any given point along the tissue, reveal collagen fibrils with near circular cross sections [83]. This lends support to the assumption that these fibrils have uniform cylindrical shape (Figure 4C and Figure 7A). In most instances, since most fibrils appear very long in scanning electron micrographs, the assumption is that the fibrils are continuous [57]; in other words, all fibrils possess lengths comparable to that of the tissue length. However, whole fibrils can be extracted by gentle mechanical means from the sea urchin ligaments [22] and while these isolated fibrils reveal a high degree of slenderness, they are shorter than the overall length of the tissue. Thus, this suggest that fibrils in the MCT can be short, rather than continuous. For further details concerning the theory of fibre reinforced composites for discontinuous fibres versus continuous fibres the reader is recommended to a recent book authored by Goh [119].

**Figure 9:**
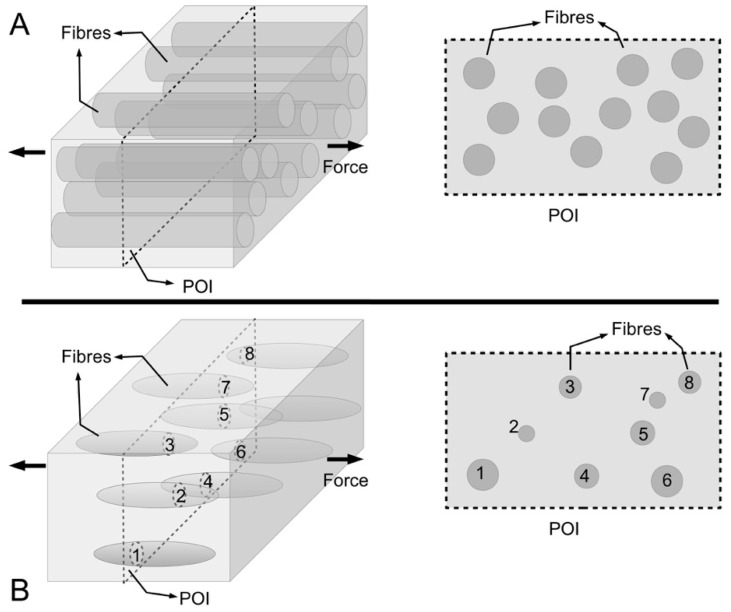
Schematics of the cross section of fibre reinforced composites. (**A**) Continuous uniform cylindrical fibre reinforced composite (**left panel**) from a 3D perspective. Corresponding 2D perspective showing the plane of interest (POI) containing the cross-sections of the uniform cylindrical fibre (**right panel**); (**B**) Discontinuous paraboloidal fibre reinforced composite (**left panel**, 3D perspective). Corresponding plane of interest (POI) showing the cross-sections of the paraboloidal fibre (**right panel**, 2D perspective). The fibres numbered, 1–8, in the 3D and 2D schematics are intended to illustrate their associations between the two views. In part (**A**,**B**), the force acting on the respective composites is in the direction of the fibre axis.

More intriguing is the shape of the fibrils. It has been pointed out that the isolated fibrils reveal naturally tapered ends [22]. This observation could be further confirmed by serial thin sections of fibrillar cross sections in intact tissues, showing how the fibrils gradually diminish in size and disappear in the ECM [22]. To further ensure that the tapered ends seen in the micrographs was not a result of imaging artifacts, Trotter and Koob contrasted the ends of the intact fibrils to the broken ends of ruptured fibrils, which shows a frayed appearance [22]. Unfortunately, it is not only difficult to track the individual fibrils serially—very few studies have been carried out to investigate this further—there is also the limitation of cross-sectional studies for determining the fibril taper. Figure 9 shows schematics of a cross section of a MCT to illustrate the latter point. If we start on the basis of fibrils with uniform cylindrical shape, and if all fibrils possess the same diameter, this can be explained by Figure 9A (right panel). However, longitudinal micrographs also reveal fibrils with varying thicknesses, and this may be illustrated by Figure 9B (right panel). However, if some fibrils have tapered ends (Figure 9B, left panel), this approach would conveniently mask the information present in the micrograph of Figure 9B (right panel).

With regards to adult vertebrate, studies have also revealed that taper exists at both ends in the fibrils in postfoetal vertebrates such as the ligaments of rats [120]. Of note, gentamicin solution, which is believed to be able to weaken the interfibrillar bonds, was used to soak the tissue so that the fibrils may be isolated from the adult vertebrate [120]. The presence of naturally tapered terminations in intact tendons from chicken embryo has also been reported using the technique of serial thin sections analysis [59]. This observation is confirmed when fibrils are isolated from chick embryonic tendons by mild mechanical means [121,122]. Thus the evidence of the presence of tapered fibrils, which possess lengths shorter than the overall length of the connective tissue, has contradicted the standard model of a tissue containing continuous uniform cylindrical fibrils.

**Figure 10:**
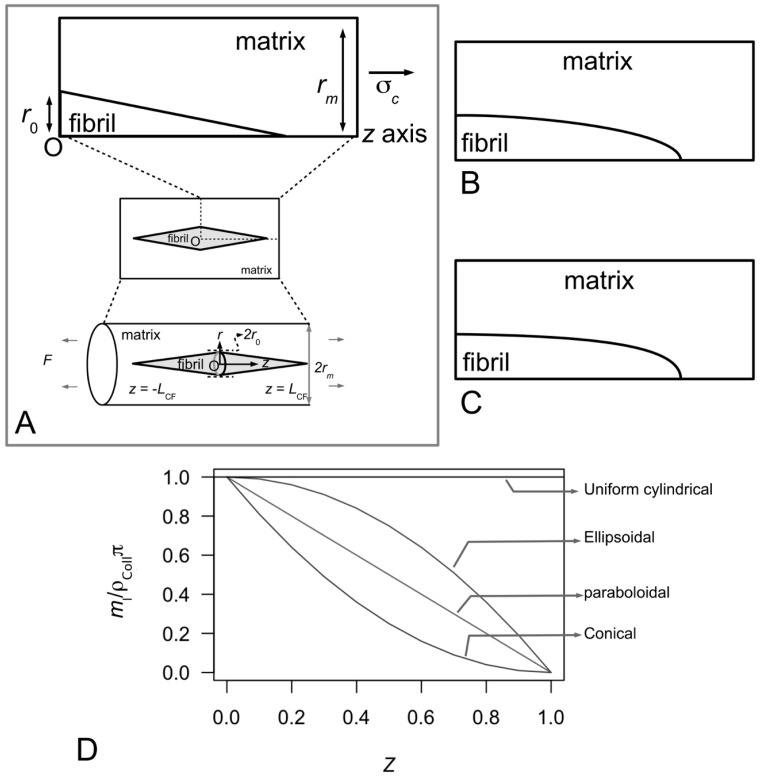
Tapered fibril reinforcing connective tissue. (**A**) A fibril with conical ends, concentrically arranged within the ECM. In this general model (see illustrations at the bottom and middle panels), the fibril possesses mirror symmetry about the fibril centre, O, and axis symmetry, which defines the *z*-axis of the cylindrical polar coordinate system, so that one-quadrant of the complete model (see illustration at the top panel) need only to be illustrated. The fibril has a radius, *r*_0_, and a half-length, *L*_CF_; *r_m_* represents the radius of the model. The stress acting on the model is represented by σ*_c_*, acting in the direction of the axis. The other fibril shapes, namely a fibril with paraboloidal ends, and an ellipsoidal fibril are depicted in (**B**,**C**), respectively. These models also adopt similar assumptions of mirror and axis symmetry developed for the conical shape so that only one-quadrant of the complete model is illustrated in the respective subfigure (**B**,**C**). (**D**) Graph of normalized fibril axial mass, *m_l_*/*ρ*π, versus *Z* from the centre to the end of collagen fibril for the respective shapes. The graphs are obtained by evaluating the respective Equations (15)–(18). Here, *m_l_* and *ρ*_Coll_ represent the collagen mass per unit length and density, respectively.

Collagen fibrils with tapered ends have also been observed in reconstituted collagen ECM—Holmes and co-workers showed that collagen fibrils generated in vitro (at 37 °C) by enzymic removal of C-terminal (i.e., the carboxyl group end) propeptides from type I pC-collagen displayed both finely tapered end and coarse end [123,124]. Figure 10A–C illustrate possible tapered profiles for a collagen fibril. Let *m_l_* be the collagen mass per unit length and *ρ*_Coll_ be the density of collagen; *m_l_* is defined as the ratio of *MN* to 5*D*, where *M* is the mass of a collagen molecule (=290 kDa), *N* the number of molecules intersecting a fibril cross-section (through an overlap region) and *D* the so-called *D* period of a collagen molecule (Figure 4A and Figure 6C). A simple analytical argument, based on Holmes and co-workers [123], results in

*m_l_*/π*ρ*_Coll_ = 1-*Z*, (15)

for a fibril that possesses a paraboloidal profile (Figure 10B). By a similar argument the relationships between *m_l_* and *Z* corresponding to uniform cylindrical, conical (Figure 10A) and ellipsoidal (Figure 10C) shapes are given by the respective equations,

*m_l_*/π*ρ*_Coll_ = 1, (16)

*m_l_*/π*ρ*_Coll_*=* 1*-Z*^2^, (17)

*m_l_*/π*ρ*_Coll_*=* −[1*-Z*]^2^. (18)

Figure 10D shows the graph of normalized mass per unit length versus fractional axial distance describing the plots of Equations (15)–(18). Thus the analytical model reveals that the *m_l_*-*Z* relationship follows a linear relationship (Figure 10D) in a fibril that has a paraboloidal shape (Figure 10B). On the other hand, the *m_l_*-*Z* plots (Figure 10D) for a fibril with conical ends (Figure 10A) and ellipsoidal shape (Figure 10C) show non-linear decreasing *m_l_* with increase in *Z*. In particular, the conical shape yields a concave profile while the ellipsoidal shape yields a convex profile (Figure 10D). Finally, we find that the uniform cylindrical shape yields an even distribution of *m_l_* independent of distance along the fibril.

To quantify the shape of these tapered fibrils, the different *m_l_*-*Z* relationships were fitted to distribution of mass as a function of axial position along the fibril derived from scanning transmission electron micrographs of reconstituted collagen (as well as embryonic tissues). It is observed that the *m_l_*-*Z* relationship that best fit the experimental data corresponds to the paraboloidal shape. Thus the mass per unit length, *m_l_*, along all fibrillar ends increases almost linearly with increase in axial distance, *z*, from the fibril end [123,125]. One important implication of these findings is related to the accretion rate, which refers to the rate of collagen being added per unit area of the fibril surface. On the basis of these findings, it is concluded that accretion rate cannot be the same at any given site of the collagen fibril [123,125]. In particular, accretion rate is likely to decrease as the fibril diameter increases [123,125].

Interestingly, so far only isolated fibrils from the MCTs of some sea urchins and embryonic tissues of vertebrates, as well as fibrils from reconstituted collagen, have been shown to possess paraboloidal ends. In most tissues the length of the fibrils is too long for even the most meticulous researcher to be able to establish the shape of the fibril unambiguously—very long fibrils are often regarded as uniform cylindrical in shape. To this end, several issues of structural interest remain unclear. First, since surface nucleation and accretion have been observed in early (tapered) fibrils, it remains to be seen if these fibrils can grow into uniform cylindrical fibrils. Second, at any given age point, what is the proportion of fibrils which are tapered and uniform cylindrical in shapes? Third, in addition to the uniform cylindrical and paraboloidal shapes, do fibrils possess other tapered shapes, e.g., conical and ellipsoidal? To this end, whether the fibrils possess the other tapered shapes is not clear but the *m_l_*-*Z* relationships derived from the analytical model predict that fibrils with tapered ends would follow a non-uniform *m_l_* distribution.

Figure 6B shows a representation of the axial packing arrangement of collagen molecules where the orientation of the respective molecules is indicated by the N-C termini. Of note, the orientation of the collagen molecule is an important consideration for understanding the overall polarity of the fibril as well as the overall shape of the fibril. At a more fundamental level, it turns out that the MCTs of echinoderms have exclusively *N*,*N*-bipolar collagen fibrils [126]—the collagen molecules change orientation at one location (5–10 *D* periods) along the fibril [124,126]. These fibrils grow (exclusively) by coordinated accretion of collagen molecules without any need for fusion of fibrils [127]. The invertebrate (embryonic) tissues may have either *N*,*N*-bipolar [120] or a mix of *N*,*N*-bipolar fibrils and unipolar fibrils [121,122]. Various end-to-end fusion events can occur which then result in a fibril with an N-C polarity or C-C polarity [122]. This is an important aspect of MCT versus vertebrate collagenous tissues and is almost always overlooked.

### 4.2. Taper in Fibrils Facilitates Even Distribution of Stress throughout the Fibril

Most information on fibre reinforced composites have been derived from studies of uniform cylindrical fibres reinforcing the composite [128,129]. As pointed out in the previous section, since fibrils in the MCT need not be uniform cylindrical in shape how does fibril shape contribute to the reinforcement of ECM in the MCT? Over the past ten years, studies on the biomechanics of collagen fibrils have applied the theory of discontinuous-fibre reinforced composites [80,81,100,104,105,106,112,130] to develop models to explain the basis of collagen fibrils reinforcement of ECM during the elastic stress transfer stage [27,80,81] and the plastic stress transfer stage [27,80,81,100]. The mechanisms associated with elastic stress transfer and plastic stress transfer have been described in Section 3.3 and Section 3.4, respectively. These models show that there are advantages for fibrils to be associated with tapered ends while the tissue is loaded in the elastic and plastic region but not when the fibrils begin to fail, e.g., by fibril pull-out [27,131]. In Section 3.3 and Section 3.4, it has been pointed out that elastic stress transfer and plastic stress transfer regulate the stress uptake in the fibrils when the MCT is in the stiff and compliant states, respectively. In this section, the discussion focuses on how fibrils of different shapes take up stress during the elastic stress transfer and plastic stress transfer stages.

**Figure 11:**
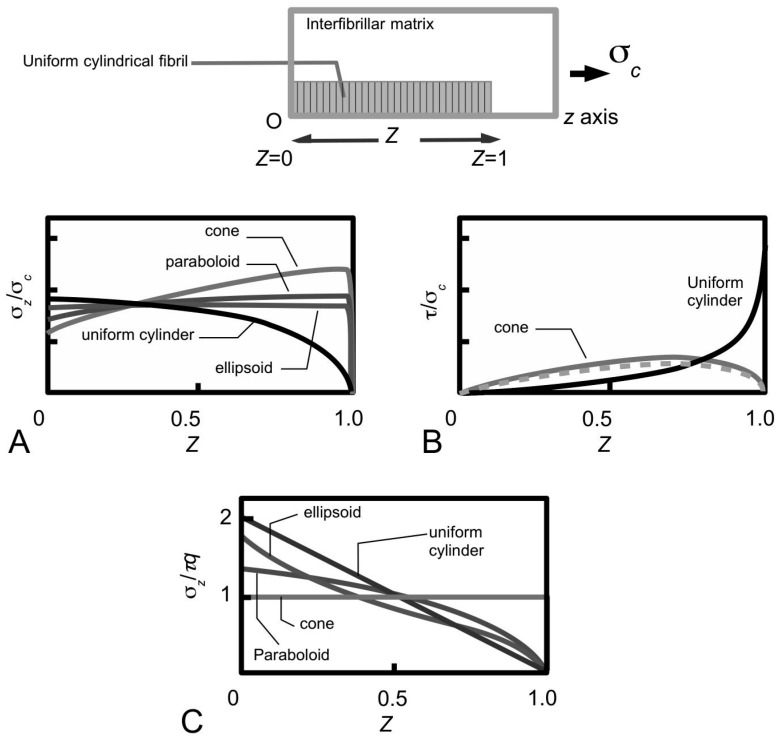
The stress distributions along the fibril axis for collagen fibrils, modelled by four different fibril shapes, namely conical ends, paraboloidal ends, ellipsoid and uniform cylinder, undergoing elastic stress transfer (**A**,**B**) and plastic stress transfer (**C**). Sketches of the (**A**) graph of normalized axial stress, σ*_z_*/σ*_c_*, [104] and (**B**) graph of interfacial shear stress, τ/σ*_c_*, [96] versus fractional distance along the fibril axis, *Z*. The results were evaluated at fibril aspect ratio, *q* = 3500, and relative stiffness of the fibril to the matrix, *E*_CF_/*E_m_* = 10^6^. (**C**) Graph of normalized axial stress, σ*_z_*/τ*q*, versus fractional distance along the fibril axis, *Z* obtained by evaluating the stress equations of the respective fibre shapes [107]. All graphs are shown for the stress plotted from the fibril centre (*Z* = 0) to one end (*Z* = 1). Here, symbols σ*_c_* represents the applied stress acting on the tissue in the direction of the fibril, τ represents the interfacial shear stress, *r_m_* represents the radius of the matrix surrounding the fibril; *Z* = *z*/*L*_CF_ where *z* is the *z* coordinate of the cylindrical polar coordinate system and *L*_CF_ represents the half-length of the fibril.

Figure 11A shows sketches of the axial stress distribution generated in the collagen fibrils during elastic stress transfer, for four different fibril shapes, adapted from the study of Goh et al. [80,81,104]. The profile of the fibril with a uniform cylindrical shape has been described previously (Figure 7B); it is plotted here for the purpose of comparison with the stress distribution profiles associated with the other fibril shape. Thus, the axial stress profile for the fibril with tapered ends is somewhat opposite to that of a fibril with uniform cylindrical shape. Here, we find that the stress in the fibril with tapered ends has a minimum value at the fibril centre, but increases non-linearly and gradually from the centre to the end, and peaks near the end point before decreasing rapidly to zero at the end. Among the tapered profiles, note that at the fibril centre, the axial stress from the conical shape yields the smallest magnitude. The axial stress corresponding to the paraboloidal and ellipsoidal fibril lie somewhat in between the conical and uniform cylindrical fibril. Figure 11B shows the graph of fibril-matrix interfacial shear stress versus axial distance. Interestingly, for the fibrils with tapered ends the fibril-matrix interfacial shear stress yield similar profile and magnitude: we observed that a minimum shear stress occurs at the fibril centre, which then increases gradually with distance, reaching a peak value at a distance somewhat halfway between the fibril centre and the end before decreasing to a minimum value at the fibril end [27,106]. (Of note, the shear stress from a fibril with uniform cylindrical shape yields a minimum value at the fibril centre but increases non-linearly to a maximum at the fibril end [106].)

Figure 11C shows the axial stress distribution in the collagen fibril for varying fibril shapes, during plastic stress transfer; the results have been adapted from the study of Goh et al. [80,81,107]. As pointed out in Section 3.4, during plastic stress transfer, the matrix becomes plastic and a weak bonding mechanism regulates the sliding action between fibrils; the shear stress generated at the fibril surface is constant of axial distance (Figure 5B). The axial stress distribution from the fibril with a uniform cylindrical shape has been described in previous section (Figure 7C) but is included here for the purpose of comparison with the results from the other fibril shapes. Thus, the axial stress distribution from the conical fibril features a uniform magnitude throughout the fibril. On the other hand, the axial stress distributions of the fibril with paraboloidal and ellipsoidal shapes vary non-linearly with distance along the fibril axis. With regards to taper, apart from the conical fibril, both paraboloidal and ellipsoidal fibrils yield stresses which peak at the fibril centre. However, the peak stress from the paraboloidal fibril is lower than that of the ellipsoidal fibril. Thus, in all the cases examined here, it appears that taper in fibrils modulates the axial stress uptake by ensuring a more uniform distribution of stress throughout the fibril.

Overall, these arguments allow us to draw general conclusions concerning the performance of the fibrils in the ECM of the MCT. It follows that taper in fibrils provide an advantage over the uniform cylindrical fibrils when the MCT is in the stiff and compliant states, where the elastic and plastic stress transfer mechanisms predominate, respectively. This advantage has to do with the lowering of the peak stress at the fibril centre. The argument is that although we would expect the elastically deforming fibril to take up stresses, high peak stress is to be avoided as this could reach the level of the yield strength of the fibril as the load acting on the MCT increases.

### 4.3. Fibrils Ought to Possess a Certain Slenderness for Appropriate Force Transmission

As pointed out in Section 1 and Figure 1A, early studies of the ligaments of sea urchins, such as *Anthocidaris crassispina* [15,16], *Echinometra lucunter* [14], *Eucidaris tribuloides* [22] reveal that the general tissue architecture of these animals are similar to those of mammalian tissue, namely a hierarchical architecture featuring collagen fibrils aligned to form bundles of collagen fibres, and bundles of collagen fibres aggregate to form fascicles within ECM [27,111]. In the sea cucumber (*Cucumaria frondosa*), collagen fibrils from the dermis of the animal are aggregated in vitro by the dermal stiparin glycoprotein [65,82]. Stiparin is mentioned in Section 3.5 for its possible role in providing bonding between the fibrils.

From these hierarchical structural concepts comes the determination of the dimensions of the fibrils. Detailed quantitative results from electron micrographs of the ligaments of sea urchin, *Eucidaris tribuloides*, reveal that the fibril possesses a minimum length of around 37 μm [126] and a maximum length of around 570 μm [22], with an average length of 225 μm [22]. These findings are comparable to other marine invertebrates such as starfish (*Asterias amurensi*), which are found to possess lengths of 20–540 μm, with a mean length of 196 μm [132]. Altogether, these findings suggest that the fibrils do not span the entire length of the MCT. As for fibril diameter, collagen fibril diameter measurements derived from electron micrographs of the spine ligament of sea urchin (*Eucidaris tribuloides*) show a minimum diameter of around 25 nm [126] and a maximum diameter of around 280 nm, with an average value of 124 nm [22]. The diameters of the collagen fibrils in the compass depressor ligament of the sea urchin (*Paracentrotus lividus*) are observed to exhibit a spread of values on the histogram with a peak frequency at 45.5 ± 19.0 nm [69]; no length measurement has been carried out [69]. These findings are also comparable to other marine invertebrates such as starfish (*Asterias amurensi*) which yields diameter ranging 10–350 nm, with an average of 136 nm [132]. Interestingly, a simple linear regression analysis reveals significant linear relationship between the lengths and diameters of the respective fibrils in the sea urchin ligament [22]. By identifying the slope of the fitted line with the *q*, it is suggested that all fibrils have a nearly constant *q* of about 5300 [22]. This slope is identified with the fibril aspect ratio. Of note, vertebrate fibrils have lengths ranging 12–30 μm and diameters ranging 40–109 nm [120]. Corresponding measurements of these individual fibrils reveal *q*s ranging 550–1025 [120]—thus it would appear that the *q* from the marine invertebrate is higher (up to one order of magnitude) than those of the vertebrate. It is not clear if the length and diameter of vertebrate fibrils also exhibit a linear relationship for the respective tissues.

Table 2 presents estimates of the fibril length, diameter and *q* based on studies of the respective tissues of marine invertebrates, namely starfish, sea urchin and sea cucumber, as well as tissues from vertebrates. As the fibril diameters of both vertebrates and invertebrates do not differ dramatically, the high collagen fibril aspect ratio from the sea urchin may be attributed to longer fibrillar length. On the other hand, one then argues that the small collagen fibril aspect ratio from the vertebrate is the result of fibrils having shorter length.

How do the collagen fibrils in the sea urchin achieve high aspect ratio? Trotter and co-workers show that the two pointed tips of a growing fibril in the spine ligaments of sea urchin (*Eucidaris tribuloides*) have similar axial mass distributions, indicating that the shape and size of the two tips remains similar throughout growth [126]. With regards to shape, these tips were paraboloidal, as evidence by the linear axial mass distributions (Figure 10D), similar to those of sea cucumber (*Cucumaria frondosa*) [133], and metatarsal tendon from embryonic chick [136]. Computer modeling reveals that the self-assembly mechanism involves independent growth by a process that produces a uniform rate of extension of the fibril tips, and lateral (i.e., radial) growth by surface nucleation and propagation [126]. Very long and slender fibrils that form the axis of the tissue can grow in length by end-to-end fusion of early fibrils involving only the C-tip (C end of the collagen molecule, see schematic in Figure 6) of a unipolar fibril [126].

**Figure 12:**
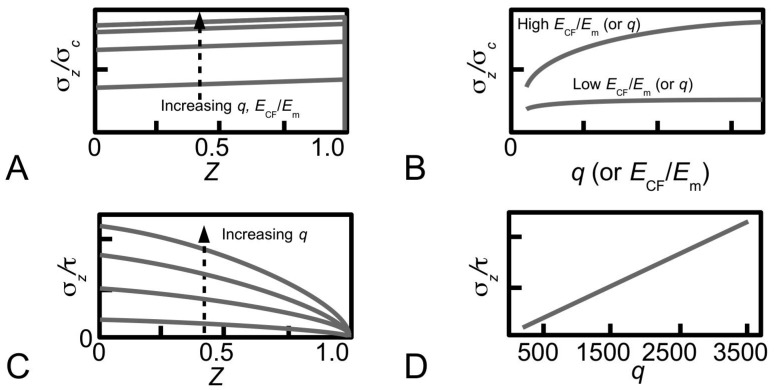
Effects of fibril aspect ratio, *q*, and ratio of moduli of the fibril to the interfibrillar matrix, *E*_CF_/*E_m_*, on the axial stress, σ*_z_*, in a fibril. Sketches of the (**A**) graph of normalized axial stress, σ*_z_*/σ*_c_*, versus fraction distance, *Z*, along the fibril and the associated (**B**) graph of σ*_z_*/σ*_c_* at the fibril centre (*Z* = 0) versus *q* (or *E*_CF_/*E_m_*) during elastic stress transfer [81]. Graphs of the (**C**) normalized axial stress, σ*_z_*/τ, versus *Z* along the fibril and the associated (**D**) graph of maximum σ*_z_*/τ (at *Z* = 0) versus *q* during plastic stress transfer; the results are obtained by evaluating the stress equation derived for the fibre with paraboloidal ends [107]. Thus, all results shown here apply to the fibril with a paraboloidal shape. The *q* values range 200 to 3500 (the arrow in part (**A**,**C**) indicates increasing *q* value). Of note, the authors of the paper describing these computer models have made clear the difficulties in meshing the model beyond an aspect ratio of 3500 and have defined a strategy that limits the analysis to within the constraints of the models; further details can be found in the reference [81]. Symbol σ*_c_* represents the applied stress acting on the tissue in the direction of the fibril and τ represents the fibril-matrix interfacial shear stress.

Szulgit has suggested that the fibrils in the ligaments may be required to possess a certain *q* in order to be able to transmit the appropriate level of force [135]. How high should the aspect ratio be in order to enable collagen fibrils in MCTs to provide reinforcement to the MCT? Goh and co-workers have investigated the stress uptake in a fibril at varying *q* by finite element analysis and analytical modelling [81]. Figure 12A shows the distributions of stress, σ*_z_*/σ*_c_*, along a fibril (paraboloidal shape), obtained for various values of *q* to illustrate the dependence of stress on *q* during elastic stress transfer [81]. For a given value of *E*_CF_/*E_m_*, it is shown that the magnitude of the stress increases when *q* increases. However, varying *q* has little effect on the profile of the stress distribution. It is also shown that the magnitude of the stress in the distribution is more sensitive to *q* at large *E*_CF_/*E_m_* than at small *E*_CF_/*E_m_*—the relationship between σ*_z_*/σ*_c_* at the fibre centre and *q* is shown in Figure 12B for two cases, corresponding to large *E*_CF_/*E_m_* and small *E*_CF_/*E_m_*. Thus, there are two important points of contention. First, the trend for each curve in Figure 12B reveals that the stress eventually converges at high *q* values. Second, the findings shown in Figure 12B predicts that *q* and *E*_CF_/*E_m_* could interact and mask the main effects. Further studies by experiment would be needed in order to clarify these points.

The dependence of the stress on *q* is also observed in plastic stress transfer. Consider the case of the paraboloidal fibril. Equation (**8**) can be solved for the stress in the fibril by substituting the expression of *r*_0_, given by *r*_0_ = [*L*_CF_/*q*]√(1-*Z*) for a paraboloid, and applying the boundary condition of σ*_z_* = 0 at *Z* = 1 [107]. This results in the following expression,

σ*_z_ =* [4/3]τ*q*√(1-*Z*). (19)

Thus, Equation (**19**) shows that the magnitude of σ*_z_*/τ in a paraboloidal fibril is linearly proportional to *q* but is independent of *E*_CF_/*E_m_*. Large values of *q* result in high magnitudes of σ*_z_*/τ during plastic stress transfer. Figure 12C shows the distributions of stress, σ*_z_*/τ, along a fibril (of paraboloidal shape), obtained for various values of *q* to illustrate the dependence of stress on *q* during plastic stress transfer [81]. Clearly, the profile of the stress distribution is not affected by varying *q*. The relationship between σ*_z_*/τ (for a given *Z*) and *q* is a linear one as shown in Figure 12D for *Z* = 0.

Thus, increasing *q* increases the magnitude of the stress uptake—the effects of *q* on the stress in the fibril in the paraboloidal case are similar to the other shapes (uniform cylindrical, conical and ellipsoidal) [104]. In other words, one good reason for the collagen fibrils in the connective tissue of the sea urchin to possess high *q* is simply because higher stresses may be generated in the fibril during the respective stress transfer processes, but the magnitude of the peak stress will always be lower than that of the uniform cylindrical fibril. Additionally, one also recalls that the stress concentration factor for a paraboloidal fibril is always lower than the uniform cylindrical fibril (Figure 11A,C). For given *q* and fibril-matrix interfacial shear stress, the extent to which the magnitude of the stress in the collagen fibril can increase would depend on the yield strength of the collagen fibril. There is a critical *q*, beyond which the stress uptake can increase to the level of the yield strength, and this could cause the fibril to yield [58,119]. In order to enable us to make useful comparison between the measured aspect ratios of sea urchin collagen fibril and the critical *q* for yielding, the latter must be known. Unfortunately, the exact value for the collagen fibril critical *q* for yielding is still not clear because we do not yet have the means of determining this parameter.

Is there any advantage for all fibrils in the spine ligament tissue of the sea urchin to possess the same *q*? Based on earlier arguments that suggests that high *q* leads to high stress uptake, if the fibrils in the tissue were to possess a range of *q*s, it then follows that a fibril which belongs to a population associated with small *q*s may not be able to take up high stress. Thus, a tissue that features a heterogeneous system of *q*s means that not all the fibrils would be able to maximize the stress uptake and the consequence is a mechanically unstable tissue. For the marine invertebrate this consequence could have severe repercussion during stress transfer, be it in the stiff (elastic stress transfer) or compliant (plastic stress transfer) states. Additionally, from the perspective of fibrillogenesis, having a fibrillar growth mechanism that self-regulate the fibril to achieve constant (high) *q* is important for the marine invertebrate.

The concept of the critical length (2*L*_crit_) for fibril fracture, borrowed from engineering fibre reinforced composites [113,119], is important for understanding how a fibril fracture [27,100]. Thus, 2*L*_crit_ is defined as the minimum length that a fibril must have for the stress at its centre to be equal to the fibril fracture strength [27,100]. For effective reinforcement, *L*_CF_ should be large but less than *L*_crit_. Analytical models have predicted that tapered fibrils have longer *L*_crit_ than uniform cylindrical fibrils, given all things being equal, i.e., *r*_0_ and τ [27]. In particular, the *L*_crit_ of a fibril with straight-tapered ends, paraboloidal ends and ellipsoidal ends are, respectively, 2, 3/2 and 4/π times longer than that of a uniform cylindrical fibril [27]. By an analogy to engineering composites [27,119], it may be argued that the longer the collagen fibrils, the tougher, stronger and stiffer will be the MCT, given all things being the same (i.e., *r*_0_ and τ). Analytical models have also predicted that a fibril with tapered ends requires less volume of collagen material to synthesize as compared to uniform cylindrical fibrils, for a given *L*_CF_ and *r*_0_ [27]—this is another good reason for the collagen fibrils in the connective tissue of the sea urchin to possess tapered ends, as well as having high slenderness (i.e., high *q*).

Can a fragmented collagen fibril grow? To answer this question, Holmes and co-workers have developed a seeding system whereby fragments of collagen fibrils can be isolated from avian embryonic tendon and added to purified collagen solution [127]. Holmes and co-workers found that the fractured ends of fibrils can act as nucleation sites for further growth by molecular accretion [117]. Two interesting findings arise from this study: (1) the surface nucleation and accretion process can result in a fibril with smoothly tapered end and (2) there is a limit to the increase in diameter as the fibril grow axially—it appears that beyond a length of 13 μm (200 *D* periods) a maximum diameter of about 600 kDa/nm is reached [127].

### 4.4. Small and Large Fibrils Have Distinct Roles in Regulating Mutability

The study of the lateral arrangement of collagen molecules is important for understanding the contribution of molecular interactions to the collagen fibril lateral size. Several models have been proposed to resolve the cross-sectional structure of collagen (type I) fibril, such as the spirally packed models and radially packed models with concentric layers of radially oriented microfibrils [137]. A radially packed model has also been developed by Silver and co-workers [138] to account for the *D* period and axial staggering of fibrils with paraboloidal ends reported by Holmes and co-workers [123]. We opined that the most important revelation concerning the structure of the collagen fibril comes from the study carried out by Orgel and co-workers [29]. From a detailed crystallographic analysis Orgel and co-workers have predicted that the collagen type I structure comprises collagen molecules arranged to form a supertwisted (discontinuous) right-handed microfibril that interdigitates with neighboring microfibrils. This interdigitation establishes the crystallographic superlattice, which comprises quasihexagonally packed collagen molecules [29]. This study is important because it effectively framed the concept of the ultra-structure of collagen fibril within a 3D space that can also account for the *D* period and staggering configuration.

Alongside with these studies include findings concerning the fibril diameters distribution in the tissue. Curiously, there are probably more investigation dealing with the quantitative analysis of collagen fibril diameters in vertebrates than in marine invertebrates. These quantitative studies evaluate the histograms of the frequency versus diameter to understand how (1) age, i.e., from development to old age [58,139,140,141], (2) symptoms, e.g., Ehlers-Danlos syndrome [46], and (3) functions of different types of tissues [139,142], influence the tissue mechanical properties. In vertebrates, while tissues from very young individuals (namely mice at birth until, e.g., 2 week old) possess diameter distribution with near-Gaussian profiles [139], the diameter distributions of tendon in mature and old individuals feature non-Gaussian profiles that may be described as bimodal [139,143,144,145] or even tri-modal [141]. It must be emphasized that the fibril diameter distribution histogram can be regarded as a quick way to provide a useful measure of the fibrillar content in the ECM. For vertebrates, since the ends are seldom encountered, this implies that the diameter of the fibril are fairly constant, at any given cross-section of the ECM, and hence the diameter distribution histogram provides a realistic picture of the distribution of the fibril sizes. This conceptual approach could be misleading. Figure 9A shows a schematic of continuous uniform cylindrical fibres reinforcing composites to model the long collagen fibrils in ECM of vertebrates; this implies that the total cross-sectional area of the fibrils is constant at any plane of interest (POI). This sectioning approach is applied to obtain transmission electron micrographs of fibril cross sections for quantitative analysis of fibril diameter in vertebrate connective tissues. Figure 9B shows a schematic of discontinuous spindle-like fibres reinforcing composites to model the short collagen fibrils in ECM of marine invertebrates such as sea urchins; this implies that the total cross-sectional area of the fibrils is not constant at any given POI. For marine invertebrates such as sea urchin, the fact that spindle-like fibrils are embedded in and reinforcing the ECM means that it is not useful to apply the sectioning approach for quantitative analysis of fibril diameter in these animals.

Several studies have been carried out to understand how fibril diameter affects the mechanical property of connective tissues such tendon and ligament [140,143,146,147]. This is analogous to the structural engineers’ concern with how steel rods embedded in concrete columns provide reinforcement to the columns, by relating the diameter of the rods to the stress uptake in the rods and cement [148]. The structural engineer would claim that it is straight-forward to evaluate the effects of diameter on mechanical properties because these steel rods can be engineered to possess uniform diameters within manufacturing errors. So far, based on the observed tissues, it is shown that the fibril diameter exhibits a spread of values that may be described as unimodal [142], bimodal [58,141] or tri-modal [141]. When attempts are made to relate the mean fibril diameter of the frequency histogram to the mechanical property, conflicting findings emerged. For instance, for a given tissue type, mechanical parameters such as structural strength (maximum force) and structural modulus of the force-displacement curve are found to be dependent on the the mean diameter [143,146]. However, the material strength (maximum stress) and material modulus of the stress-strain curve are not dependent on the mean diameter [143,146].

Goh and co-workers have carried out a study to rationalize the discrepancies using tail tendons from a mouse model; this has resulted in two key papers that hypothesize that (1) the stress-driven parameters that contribute to elasticity and fracture are dependent on the collagen fibril volume fraction, governed by the rule-of-mixture [40], and (2) the strain energy-driven parameters that contribute to resilience and fracture are dependent on the fibril diameter of the respective modal distribution, govern by fibril shear sliding theory [58]. (The approach to test these hypotheses was to apply them to tissue of varying age groups, in parallel with measurements of the fibril cross-sectional parameters (area, diameter) using transmission electron microscopy.] Without loss of generality, the findings from this study have allowed for drawing general conclusions concerning the MCT. Thus the magnitudes of the strength [40], stiffness [40], fracture toughness [58] increase linearly with increases in fibril volume fraction [40], in accordance with the rule-of-mixture for strength and stiffness [119,129]. Unfortunately, fracture toughness has a more complex nature than the strength and stiffness parameter, and consequently, the rule-of-mixture cannot be applied to relate the fracture toughness parameter to the fibril cross-sectional area [129]. How then can one evaluate the relationship between the fracture toughness and the collagen fibril structural parameter? Goh and co-workers proposed that the fracture toughness of the tissue is the sum of the nonessential energy (*u_E_*), and the essential energy (*u_F_*) according to the principles of essential work of fracture of a fibre composite [58]. The *u_E_* is said to contribute primarily to tendon resilience (regulated by fibrils undergoing elastic deformation); the *u_F_* is said to contribute primarily to the resistance of the tendon to rupture (regulated by fibril rupture, leading to defibrillation and rupture of the interfibrillar matrix, Figure 8) [58]. By evaluating the frequency histograms of diameter for all age groups using the minimal number of normally distributed subpopulations, the mean fibril diameter of the respective subpopulation may be linearly added—weighted by coefficients that depend on the fibril diameter, fibril strength and interfacial shear stress—to give the *u_E_* and *u_F_* [58]. Using the mouse tail tendon as a tissue model, the minimal number of normally distributed subpopulations is found to be equal to two across all the age groups [58]. For the purpose of this discussion, we then let the smaller and larger mean diameters be *D*_D1_ and *D*_D2_, respectively. Accordingly, the overall effect of fibril diameter on *u_E_* and *u_F_* may then be assessed by a multiple regression analysis [58]. It follows that increases in the resilience energy are associated with decreases in *D*_D1_ and increases in *D*_D2_ [58]. On the other hand, the energy to resist fracture is associated with increases in *D*_D1_, but independent of *D*_D2_. Thus, the fracture toughness argument emphasizes that at the physiological level, age variation in the fracture toughness is the result of changes in the energy for resilience and resistance to fracture; these two energy parameters are in turn influenced by the structural changes at the fibrillar level. It follows that small and large fibrils have distinct roles in the stiff state and only the small fibrils have a role in the complaint states, and hence lending to the mutability properties.

These arguments to relate the fibril structural parameters to mechanical properties have enabled us to draw general conclusions about the structure-function relationship that may be applicable to MCT. First, it is likely that the strength and stiffness of MCT depend on the collagen content (i.e., fibril volume fraction) in the ECM. Second, since the *u_E_* parameter is essentially associated with loading from the initial point until the end of the elastic region of the stress-strain curve, it is likely that the small and large fibrils have distinct roles in regulating mutability. While it is not clear why these fibrils are bestowed with distinct roles, the results suggest that the interplay of small and large fibrils help the tissue responds to external loads by absorbing the appropriate level of strain energy to achieve resilience (elastic stress transfer) and to resist fracture (plastic stress transfer).

### 4.5. Fibril Slenderness and Relative Stiffness Interplay to Lower Stress Discontinuity in Fibril Interaction

The CA of the sea urchin can undergo deformation without buckling [14]. To illustrate this point, Figure 1B depicts two positions, i.e., X and Y, that the spine can assume. Each position would involve a change in length of the CA [14] but the ligament is not known to buckle at position Y [14]. One way to ensure that the ligament deforms to the desired length is for the collagen fibrils, either singly or in bundles, to undergo relative axial and lateral displacements without resulting in an appreciable change in the fibril length. Thus two key features stand out with regards to the MCT. The first feature, that the collagen fibrils in the connective tissue of the sea urchin are discontinuous has already been pointed out in previous sections. We recall the length distribution of isolated fibrils in Table 2 and the demonstration of tapered ends as explained by a schematic in Figure 9B. The second feature is the physical ease of extracting and isolating intact fibrils from the ligament, by gentle mechanical procedures [22]. These fibrils can retain their native structure over long duration in fluid suspensions [22]. The second feature suggests that the bonds that normally hold the fibrils in the ECM are labile but the collagen fibrils are very stable [22]. While the precise fibril-fibril sliding mechanism is not clear, these features suggest that fibril-fibril sliding could contribute predominantly to the tissue deformation as proposed by Smith et al. [14] and by Hidaka and Takahashi [15].

**Figure 13:**
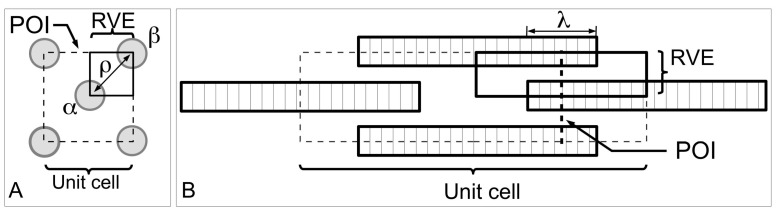
Model of ECM containing short (uniform cylindrical) collagen fibrils arranged in the square-diagonally packed configuration. (**A**) A cross-sectional (plane of interest, POI) view; (**B**) The longitudinal view of the unit cell. In part (**A**), α refers to the primary fibril of interest; surrounding the α fibril are the β (secondary) fibrils of interest. Here, RVE represents representative volume element; λ and *ρ* represent the fibril-fibril axial overlap distance and the centre-to-centre lateral separation distance, respectively.

One finds that when a load acts on the MCT, the fibrils are pressed against the interfibrillar matrix. As the load increases, the magnitude of the component of the resultant force acting on the fibril that is associated with friction—i.e., the normal force—at the contact surfaces also increases [119]. Altogether, these contact forces regulate the fibril stretching and sliding (relative to the matrix); the matrix may be regarded as responsible for transmitting stress to the fibril [119]. Nevertheless, how does a fibril take up stress during fibril-fibril sliding in the MCT? Figure 13 shows a model of ECM in the MCT to help understand the mechanism of fibril-fibril sliding. Here, we shall draw some general conclusions from a previous study on fibre-fibre interaction that used a unit cell approach to establish the stress uptake in a fibril during fibril-fibril sliding. Figure 13A,B show a central (α) and peripheral (β) fibrils for illustrating the unit cell model at two different views. The unit cell is further divided into representative volument elements (RVEs) (Figure 13A,B). This model can be used to evaluate the elastic stress transfer process in MCT with the following simplifying assumptions, namely (1) strong adhesion is present at the fibril-matrix interface; (2) as the interfibrillar matrix around the fibril deforms elastically, shear forces will develop at the fibril-matrix interface; (3) in response to the shear action the fibril deforms elastically and generates an axial tensile stress. Altogether these assumptions are consistent with the “shear-lag” approach (Section 3.3).

**Figure 14:**
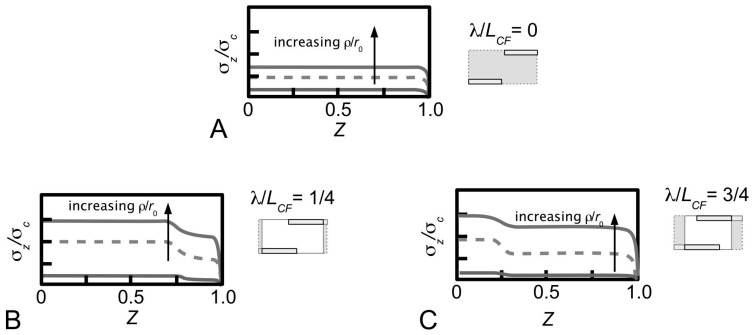
Fibril-fibril interaction. Sketches of the graph of axial tensile stress, σ*_z_*/σ*_c_*, in a fibril versus distance, *Z*, along the fibril axis (where *Z* = 0 and 1 correspond to the fibril centre and end, respectively) at (**A**) λ/*L*_CF_ = 0; (**B**) λ/*L*_CF_ =1/4 and (**C**) λ/*L*_CF_ = 3/4 for the uniform cylindrical shape at varying fibril-fibril separation distance, *ρ*/*r_o_*, adapted from the report of Mohonee and Goh [130]. Insets (right of each graph) show representative volume elements (RVEs, Figure 13) of fibrils embedded in the matrix at different overlap distance. In the report of Mohonee and Goh, all results have been obtained by setting the ratio of the stiffnesses of the fibril to the matrix, *E*_CF_/*E_m_*, equal to 10^2^ (“low”) and the fibril aspect ratio *q* = 650 (“high”). Symbol σ*_c_* represents the applied stress acting on the tissue in the direction of the fibril, *L*_CF_ represents the half-length of the fibril and *r*_0_ represents the fibril radius (for the tapered fibril, this refers to the radisu at the fibril centre).

According to finite element analysis [130], one arrives at the stresses, σ*_z_*/σ*_c_*, in the fibril at varying fibril-fibril axial overlap distance (λ) and the centre-to-centre lateral separation distance (*ρ*) as a function of axial distance along the fibril, for the case of the uniform cylindrical shape (Figure 14). When the neighbouring fibrils do not overlap, i.e., the α and β fibrils in the unit cell do not overlap along the axes (λ/*L*_CF_ = 0), the stress uptake within the α fibril is modulated by the α fibrils from the adjacent unit cells (Figure 14A). Thus, σ_z_/σ*_c_* is maximum at the fibril centre (*Z* = 0) which then decreases steadily to zero at fibril end (*Z* = 1). The stresses are very evenly distributed throughout the bulk of the fibril (except at the fibril end) at high *q*. In contrast, the stresses show appreciable decrease in magnitude with increasing *Z* at low *q* values (results not shown). It must be pointed out that the stresses at high *E*_CF_/*E_m_* feature even distributions throughout the bulk of the fibril (except at the fibril end) for both cases of high and low *q* (results not shown). This is important because it suggests that the interplay of *E*_CF_/*E_m_* and *q* predominates in the fibril-fibril interaction. The result is higher axial stress even when the fibrils in the immediate vicinity are not overlapping with the primary fibril.

An important implication of fibril-fibril interaction is the generation of stress discontinuity when neighbouring fibrils overlap, i.e., when the α and β fibrils overlap along the axes (λ/*L*_CF_ > 0) (Figure 14B,C). Here, the stress uptake within the α fibril is modulated by the β fibril, as well as by the α fibrils from adjacent unit cells. This leads to higher σ*_z_*/σ*_c_* throughout the fibril compared to the case when overlap is absence (λ/*L*_CF_ = 0). The stress discontinuity is described as an abrupt (stepwise) change in the σ*_z_*/σ*_c_* distribution, which is most pronounced at high *q* (Figure 14B,C), but somewhat less so at low *q* (results not shown). Additionally, the stress discontinuity (i.e., a sudden drop in stress) results in higher σ*_z_*/σ*_c_* at the non-overlapped region and lower σ*_z_*/σ*_c_* at the overlapped region (Figure 14B,C). It must be emphasized that the exact position of the stress discontinuity varies with the extent of overlap. Indeed, increasing λ/*L*_CF_ displaces the discontinuity to the fibril centre. Interestingly, when axial overlap occurs, the magnitude of the stress at a given location in the fibril (within the overlap region) appears to be independent of the extent of the axial overlap, regardless of the point within or outside the overlap region. Thus, it is important to note that no further advantage (i.e., higher stress uptake) may be gained from increasing the overlapping region.

With regards to the lateral separation distance, one notes that the extent of the step-wise change in the stress magnitude is dependent on the lateral separation, i.e., *ρ*-dependent, as well as *q*. In general, large *ρ*/*r*_0_ leads to small step-wise change in the stress uptake. This implies that the influence of the β fibril decreases with increasing *ρ*/*r*_0_ (Figure 14). More importantly, the stress discontinuity disappears at high *E*_CF_/*E_m_*, regardless of *q*. Additionally, increasing the fibril-fibril separation distance has the effect of increasing the stress magnitude in the fibril. Thus the larger the fibril-fibril lateral separation distance the higher is the stress in the fibres. Secondary to this effect is the asymptotic increase of the stress magnitude to a steady value at large fibril-fibril separation (results not shown). Of course, these conclusions apply regardless of the extent of fibril-fibril overlap; in other words, these conclusions apply when λ/*L*_CF_ = 0 or λ/*L*_CF_ > 0. Clearly the case of λ/*L*_CF_ = 0 implies that no axial overlap occurs, but this asymptotic increase in the stress magnitude with increasing fibril-fibril separation should not be interpreted to imply that the nearest (β) fibrils has no effect on the σ*_z_*/σ*_c_*. In this case, the effect on the stress with varying fibril-fibril separation distance is predominated by two factors: one, the stress field arising from the interactions with the nearest (β) fibrils where the tips of these fibrils are in line with the tip of the α fibril, and two, the effects of the bulk ECM surrounding the α fibril.

At any given λ/*L*_CF_ the largest *ρ*/*r*_0_ beyond which the effect of fibril-fibril interaction (i.e., directed by the nearest (β) fibres) diminishes may be determined from a plot of σ*_z_*/σ*_c_* (at a fixed *Z*) versus *ρ*/*r_o_*. To assess for convergence, note that at a given value of *q* at low *E*_CF_/*E_m_*, a plot of σ*_z_*/σ*_c_* (namely *Z* = 0, as the reference point) versus *ρ*/*r*_0_ shows that σ*_z_*/σ*_c_* increases rapidly with increase in *ρ*/*r*_0_ (graph not shown). More importantly, beyond a critical *ρ*/*r*_0_, σ*_z_*/σ*_c_* converges to a steady value.

The fibril with uniform cylindrical shape represents one extreme of possible regular profiles. To the best of our knowledge, no attempts have been made to model fibril-fibril sliding to study the stress distribution of fibrils that possess tapered ends, such as conical ends, paraboloidal ends and ellipsoidal shape. Nevertheless, this issue has been targeted for investigation in future studies.

Finally, it is important to emphasize that the sliding action of collagen fibrils is not to be confused with the sliding action of collagen fibres. In the latter, this has important implications on the microscopic crimp in MCT [66]. Crimp is not known to be present in the CA ligament of the sea urchin [14] (Section 2.1) but recently, it is thought to be present in the compass depressor ligament of the sea urchin, due to the observed toe-to-heel feature on the stress-strain curve of excised tissues tested to rupture at low loads [66], which is typical of most vertebrate soft connective tissues where crimp has been observed [115,149,150]. At initial loading when the load is low, i.e., within the toe-to-heel region, as the tissue strain increases the sliding of collagen fibres eventually results in the extinction of the wavy crimps [66]. Crimp is believed to originate from the contraction of cells (e.g., fibroblasts) residing on collagen fibres [151]. The mechanics of the contraction of the cells results in the buckling of the fibres [151]. Crimp can exist as early as during embryonic development in vertebrate connective tissue [151] but whether this applies to the MCT is not entirely clear. The above arguments used to lend to support to a mechanical basis for crimps in MCT suggests that crimp is analogous to a mechanical damper [152]. Consequently, crimp is hypothesized to (1) absorb energy during elastic stress transfer [27], (2) enable the tissue to recoil when the load is removed [153,154,155], and (3) absorb energy generated in shocks [115,153,154,155]. According to the load-sharing concept in fibre reinforced composite [119], it follows that the force generated within the collagen fibres for the stretching/contraction is proportional to *E*_CF_/*E_m_*. Consequently, one could expect that the larger the *E*_CF_/*E_m_* the higher is the force generated to stretch/contract the fibres. Estimates for *E*_CF_/*E_m_* ranges 10^3^–10^6^ (Table 3) [80,81]. To what extent should crimp be exploited for ECM-DT, or even synthetic collagen fibrils in a synthetic matrix is not clear but the arguments of previous studies suggest that crimp presents some advantages for the tissue to stretch/contract, aided further by virtue of the high *E*_CF_/*E_m_*.

### 4.6. Water and Charged Species within the Interfibrillar Matrix Contributes to the High Poisson Ratio of MCT

This section is intended to examine the key ECM components in the interfibrillar matrix that contribute to the mechanical properties of interfibrillar matrix. The interfibrillar matrix is believed to play an important role in fibril-fibril sliding, by an analogy to engineering fibre reinforced composites [119]. This section is concerned with the physical properties of the key constituents that contribute to fibril-fibril sliding. In this analogy, one finds that when a load acts on the MCT, the fibrils are pressed against the interfibrillar matrix [119]. As the load increases, the magnitude of the component of the resultant force acting on the fibril that is associated with friction—i.e., the normal force—at the contact surfaces also increases [119]. Altogether, these contact surface forces regulate the fibril stretching and sliding (relative to the matrix) while the matrix may be regarded as responsible for transmitting stress to the fibril [119].

For simplicity, the interfibrillar matrix of the MCT may be regarded as composed of water and charged species [61]. Since the ions are dissolved in the water of the interfibrillar matrix, the ions and the water could be responsible for regulating the fibril-fibril sliding action that results in the transition between the stiff and compliant states [14,16,66]. In the compliant state, as well as in the standard state, the fibril-fibril spacing of the compass depressor ligaments of the sea urchin is consistent with the length of the filament connecting between two fibrils, i.e., of the order of 50 nm; the spacing is much smaller in the stiff state [69]. The smaller fibril-fibril spacing in the stiff state, as compared to the compliant state, suggests that the water and charged species in the interfibrillar materials could also be displaced out of the interfibrillar space. Ultimately, the effectiveness of these ions for regulating the fibril-fibril sliding action would depend on the type and composition of the ions [14,16,66].

The initiation and propagation of fibril-fibril sliding occurs through the alterable interactions of charged species within the interfibrillar matrix—the mobility of the charged species depends on the amount of water [61]. Of note, the limit beyond which alterable interactions terminate is related to plastic stress transfer (Section 3.4). Results from FTIR and Raman spectroscopy indicate that water is largely exuded when the sea urchin compass depressor ligament changes from the compliant to the stiff states [61]. Additionally, water exudation is also observed in other MCTs such as the dermis of the sea cucumber when the tissue undergoes a change in the mechanical state [74]—by studying the respective mechanical states corresponding to compliant, standard (normal) and stiff, it has been found that the mass and volume decrease by 15% when the dermis changes from the normal state to the stiff state by mechanical stimulation and by chemical stimulation with potassium-rich seawater [69]. To this end, it is believed that the mechanisms responsible for the transition from the soft state to the standard (normal) state, and that from the standard (normal) to the stiff state, are different [74].

The water molecules exuded from the ECM include those bound previously to glycosaminoglycans side-chains mediated by electrostatic forces as well as those in free state. For the former water molecules, the electrostatic interaction may be displaced by stronger interactions involving tensilin (a collagen-fibril binding protein released from a juxtaligamental-like cell [66,70]) (Section 3.4), thus enabling the dermis to change from the compliant to the normal state and into the stiffened state [74]. Additionally, ECM components in the interfibrillar matrix that are involved in mediating the process of water exudation could also be released from the ECM [158]. Further study shows that the exudation of the water molecules and the interplay between the water molecules in the bound state and in the free state result in a water concentration gradient in the ECM, as observed in the stiffened state [61].

Water exudation can also occur in vertebrate tissues such as articular cartilage [159], mammalian tendon [160,161], and intervertebral disc [162] when these tissues are deformed. Haverkamp and co-workers found that when bovine pericardium is loaded, water exudation from the ECM could be a contributory factor to the high Poisson’s ratio (>0.5) of the tissue at strains as high as 0.25 [156]. Goh and co-workers pointed out that similar changes to the ECM components, in the interfibrillar matrix, resulting in an increase in the interaction energy between fibrils via collagen-bound proteoglycans, could happen during freezing [163]. Additionally, changes to the long-range order of radially packed collagen molecules in fibrils could also happen and this could contribute to fibril rupture at higher stresses [163].

The distribution of water in the ECM depends on the mode of the loading, e.g., uniaxial loading and mechanical relaxation [164]. Although it is still not clear how the microenvironment of the interfibrillar matrix facilitates the respective mode of loading, the interplay among macroscopic related factors, namely the osmotic pressure and the anisotropy, have been identified as possible contribution to the exudation of water and other ECM components [165]. In particular, the osmotic pressure contributes to the slackness of the tissue before stretching, resulting in tissue swelling; consequently when the tissue is straightened, as water molecules are forced out from the microenvironment of the interfibrillar matrix, an appreciable decrease in the volume of water occurs [156]. Both the osmotic pressure and the fibril direction are expected to contribute to tissue anisotropy, resulting in a Poisson’s ratio that is greater than 0.5 from the axial to the lateral direction [165].

The issue of tissue anisotropy has been addressed by Haverkamp and co-workers recently. Haverkamp and co-workers inferred that the high Poisson’s ratio of the tissue could be the result of a high Poisson’s ratio of the collagen fibrils [156]. Analysis of the small angle X-ray scattering patterns of a deforming bovine pericardium reveals that the collagen fibril Poisson’s ratio, identified with the ratio of collagen fibril width contraction to length extension, is 2.1 ± 0.7 for a tissue strained to 0.25 [156]. Since the Poisson’s ratio of the collagen fibril is greater than 0.5, this study shows that the volume of individual collagen fibrils decreases with increasing strain [156]. The change in volume in the fibril during fibril deformation implicates that a proportion of water or charged species could reside in the fibrils and that these are also exuded during stretching. How does the interfibrillar matrix contribute to the high Poisson’s ratio of the tissue? For simplicity, one may model the ECM as comprising collagen fibrils which are uniformly distributed throughout the length of the tissue such that the area fraction of the collagen fibrils at any given cross sections along the length of the tissue remains unchanged. To order of magnitude, the Poisson ratio of the tissue can be estimated according to the rule of mixture for Poisson’s ratio [128],

*v_c_* = *v*_CF_*V*_CF_ + *v_m_V_m_*, (20)

where *V*_CF_ and *V_m_* are volume fractions of the collagen fibrils and matrix, respectively, satisfying the condition of *V*_CF_ + *V_m_* = 1. By considering the upper and lower limits of *V*_CF_ to be 0.8 and 0.2, respectively [40], the upper limit of *v*_CF_ ~2 [156], and the upper limit of *v_c_* ~4 [157], the *v_m_* is found to range from 3 to 18. The estimated upper limit for the interfibrillar matrix is consistent with a material that exhibits very large change in volume during deformation.

How does the exudation of water and other ECM components from the ECM affect the mechanics of stress uptake in the fibril? According to a study of the effect of *E*_CF_/*E_m_*, which represents the ratio of the stiffnesses of the fibril (*E*_CF_) to the interfibrillar matrix (*E_m_*), on collagen fibril stress uptake, it has been predicted that the higher the *E*_CF_/*E_m_*, the larger is the magnitude of the axial stress generated in the fibril (Figure 12A,B) [81]—the axial stress uptake is more sensitive to *E*_CF_/*E_m_* than *q* [81]. As high *E*_CF_/*E_m_* corresponds to an interfibrillar matrix in a compliant state while low *E*_CF_/*E_m_* corresponds to an interfibrillar matrix in a stiffened state (which could be the result of the exudation of water and other ECM components), this suggests that the stress uptake in a fibril is higher when the MCT is in a compliant state than stiffened state.

With regards to the interfibrillar shear stress, τ, there has been several attempts to measure τ in order to provide insights into the nature of the interfibrillar matrix. Table 4 lists estimates of the interfacial stress stress derived from different studies. Recently, a novel notch tensile testing approach have been used to estimate τ in a rat tail tendon undergoing deformation [109]. Based on data derived from axial stress gradients along the tissue sample length, τ is shown to have a value of 32 kPa [109]. This estimate is comparable to the interfibrillar shear stress predicted by a multiscale model of tendon fascicles [90], as well as a strain energy density model for the resilience and fracture toughness of soft tissue in the presence of ageing [58]. In particular, the strain energy density model predicts, to order of magnitude estimates, that τ ranges 1–100 MPa [58]. A separate test using optical tweezers to measure the direct forces of rupture between two decorin proteoglycans [166] led to an estimated value of 7.5 kPa for τ [58]. Of note, targeted disruption of the decorin gene, resulting in decorin-deficiency in the tissues, yields uncontrolled lateral fusion of collagen fibrils as well as lower tensile strength (force) of the tissue, compared to native tissues [46]. Altogether these findings are important as they support the arguments that the interfibrillar matrix can transmit stress to the fibrils through interfibrillar shear. According to the basic principles of mechanical engineering, the ECM components, possibly proteoglycans (Figure 4), in the interfibrillar matrix of MCTs would have to provide cross-linkages between the fibrils and between the fibril and the matrix in order to facilitate the transfer of stress from the matrix to the fibrils. Computational models for linking the ECM components (in the interfibrillar matrix) to the fibrils, such as those developed by Redaelli and co-workers [45], may be able to provide deeper understanding for how the ECM components in the interfibrillar matrix transmit load between fibrils.

However, the contribution of the macromolecules to the shear action of the interfibrillar matrix in response to an external load is unclear because of controversy surrounding some of the ECM components that have been identified. In earlier works, Goh and co-workers [27,58,80,81] as well as many others [45,167,168] have attributed the contribution to the shear action to the interactions of glycosaminoglycan chains in the interfibrillar matrix with glycosaminoglycan attached to the core protein of small leucine-rich proteoglycan (e.g., decorin), or between glycosaminoglycan of the proteoglycan on adjacent fibrils. However, recent studies are challenging the importance of the proposed mechanical role of proteoglycans in these tissues. These studies focus on selective removal of glycosaminoglycans from the tissue via enzymatic depletion of the glycosaminoglycans using chondroitinase ABC [11,66,79,169,170]. The treated tissue have been subjected to dynamic viscoelastic tensile tests to measure the storage modulus [169], or even simple tensile test to rupture to measure strength and stiffness of the tissue [11,170] and to specific strain level to evaluate the changes in the periodic banding of the collagen fibrils (D-period) or fibril diameter [79]. While results from most mechanical parameters yielded no significant difference with the controls, for the specific strain level, it is observed that the strains generated in the fibril in tissues without glycosaminoglycans are higher than those from the controls [79]. Thus the results from the strain measurement suggest that glycosaminoglycans may serve to reduce (not increase) stress transmission between fibrils [79]. These observations could direct attention to other ECM components, namely the FACIT (i.e., fibril associated collagens with interrupted triple helices) family of molecules (collagen types XIV and XII) [25], emu1/emu2 [171] and the COMP (i.e., cartilage oligomeric matrix protein) [172] as pointed out by Szczesny and co-workers [90]. Clearly, new studies should be carried out to investigate the mechanical properties of other ECM components, because some of these could be possible candidates for having a role in the interfibrillar stress transfer mechanism. This review proposes that the news studies should consider a force-spectroscopic analysis of the molecular interaction effects of all possible ECM components—inspired by the study carried out for decorin-decorin interactions [166], alongside arguments that involve simple order-of-magnitude estimates [58]—to eliminate the suspicion on the ECM components that do not contribute to the transfer of stress from the interfibrillar matrix to the fibril as well as fibril-fibril interaction. Further discussion is out of the scope of this review but this has been targeted for future investigations.

## 5. Framework for Collagenous ECM Mechanics, Prospects and Challenges for Scaffold Design

A complete understanding of the structure-function relationship of collagenous tissues is the central goal to elucidate the mechanical properties of these tissues. In this regard, it is important to be able to see the forest for the trees, to use an apt expression. To this end, a simple framework for mapping the different mechanisms has been proposed as a systematic approach to tackle this goal [27]. These mechanisms are concerned with the stress uptake in the structural units reinforcing ECM at the respective levels of the hierarchical architecture of connective tissues. The framework enables (1) comparison of these mechanisms, (2) predictions based on the interconnection of these mechanisms and (3) identification of new mechanisms and pathways [27]. Here, we will show how the mechanisms highlighted in this review for the MCT may also be framed within this framework (Figure 1).

This paragraph and the following paragraph, are concerned with a technical description of the framework. Consider initially the macroscopic mechanical response of the MCT described by a typical stress–strain curve in tensile loading mode. Based on the arguments presented in earlier sections of this paper (Section 3 and sub-sections therein), it follows that there are five categories of mechanisms involve in regulating the stress-strain curve. The role of each category are reflected in the respective regions, namely toe-to-heel, elastic deformation, yielding, plastic deformation and rupture. The regions of elastic deformation and of plastic deformation correspond, respectively, to the stiff state and the compliant state; the region leading to rupture corresponds to autotomy. A list of known mechanisms under each category can be found, at the respective length scale. Five levels, identifying the respective length scales, are shown here. These are, from the higher level to the lower level, the macroscopic tissue, collagen fibre, collagen fibril, microfibril and the collagen molecule at the atomic/molecular level. Without going into the details of each mechanism, at this juncture it is important to emphasize the connectivity between each mechanism at the highest level to the next level, as shown by the vertical lines. The connectivity indicates possible exchange of input parameters between the respective levels. The connection from the lowest to the highest level may be termed as a mechanical pathway, by an analogy to the signal pathway concept.

The mechanisms identified in each mechanical pathway typically involve stretching, of the key components, in combination with twisting and bending, as well as relative sliding. For convenience, the term used to refer to the respective length scales correspond to the key components, namely collagen fibres, fibrils, microfibrils and molecules. Additionally, with regards to influencing the respective mechanisms, one notes that the common structure-related properties are: *q* (encompassing length and diameter), orientation and spacing between components. The common function-related properties are: stiffness, strength, strain at fracture, and fracture strain energies. Generally, all properties are involved in:

(1) decrimping, elastic deformation by fibre–fibre sliding, fibre yielding, plastic deformation, and fibre defibrillation or rupture at the collagen fibre level;
(2) the extinction of fibril waviness, uncoiling of the fibril-associated proteoglycan glycosaminoglycan side-chains, elastic stress transfer, intermediate modes (such as interfibrillar matrix cracks, partial delamination of interface between the interfibrillar matrix and fibril, and plastic deformation of the interfibrillar matrix), plastic stress transfer (with complete delamination of interface between the interfibrillar matrix and fibril), rupture of interfibrillar matrix, and fibril rupture and fibril pullout at the collagen fibril level;
(3) the straightening of microfibrils, microfibrillar sliding and realignment of microfibril from its supertwist, exudation of water and solutes from the intermicrofibrillar matrix, microfibrillar stretching, disruption of microfibril-microfibril interactions and microfibril rupture at the microfibril level;
(4) the straightening of kinks on the molecule, molecular stretching (involving axial deformation of the backbone, uncoiling the helices and helix–helix sliding) and intermolecular shear (nucleation of slip-pulse), and disruption to the intramolecular cross-links and intermolecular cross-links at the collagen molecular level.

What information can we derive from the respective mechanical pathways of the MCT? For the purpose of this discussion, this paragraph will highlight the mechanical pathway associated with elastic deformation. At the tissue level, as this regime corresponds to low applied loads, the deformation of the tissue in response to the applied load is analogous to a stretched spring; for simplicity, the stress-strain behaviour conforms to a linear relationship [173]. One could expect that the MCT relaxes and assumes its original state upon removal of the applied load. (In order for the MCT to continue to be structurally effective, one then expects that any residual stress generated on the return path to be minimized.) At the collagen fibre level, the contribution to deformation could come from fibre-fibre sliding as well as fibre stretching. At the next lower level collagen fibril-fibril sliding, as well as fibril stretching, underpin collagen fibre stretching. The stress uptake in the fibril is governed by the well-known elastic stress transfer which is used to explain how the MCT takes up load in a stiffened state (Section 3.3). In order for the fibril to be able to deform, at the next lower level one finds processes for regulating microfibrillar sliding as well as the realignment of the microfibril supertwist, and stretching, in the direction of the load acting on the fibril. The deforming microfibril is the result of sliding and stretching of collagen molecules (Section 3.3) and the intermolecular shear.

What are the gaps in the framework that have to be addressed before it can be applied to the development of ECM-DT? From the fundamental perspective, many of the proposed mechanisms along each mechanical pathway of this framework contain contentious issues that are still far from clear and could be subjects for further investigation. To begin, the fibril-fibril sliding action addresses the ECM component, namely glycosaminoglycan, that contributes to the mechanical response of the interfibrillar matrix. Currently the exact role of the ECM component is still unclear (Section 4.5). The second issue addresses the proportion of fibrils with the respective fibril shapes in the MCT. Currently it is unclear if the MCT contains a heterogeneous population of different fibril shapes or a homogeneous system of fibrils of the same shape (Section 4.2). Third, while plastic stress transfer is identified as the loading regime for the compliant state (Section 3.4 and Section 4.6), is this the limit beyond which alterable interactions terminates for the MCT? Additonally, what then are the implications for a fail-safe mechanism? Fourth, with regards to the concept of mutability, we have only provided a simple explanation (Section 2.1) with regards to fibril sliding mechanics, which is regulated by a nervous system. While a detailed discussion of the mechanism of mutability dictated by the control of a nervous system is out of the scope of this review, we are also not aware of any studies that purposefully adapted the nerve-control system to the ECM mechanics framework. Thus, it is difficult to see how we could comment on the concept of mutability in the context of the ECM mechanics framework, in this review. Last but not the least, as pointed out in Section 1, the fibril-forming collagens in the connective tissues of invertebrates such as sea urchin, as well as sponge, may have more varied structural features than those of the standard fibrillar vertebrate collagens, e.g., triple helical domains of varying lengths [32,33]. How the variability in the fine structure of these fibril-forming collagens (molecular level) affects the mechanical properties of the fibrils (fibrillar level) and, consequently, the bulk tissue is not clear and could be a subject for further investigation.

**Figure 15:**
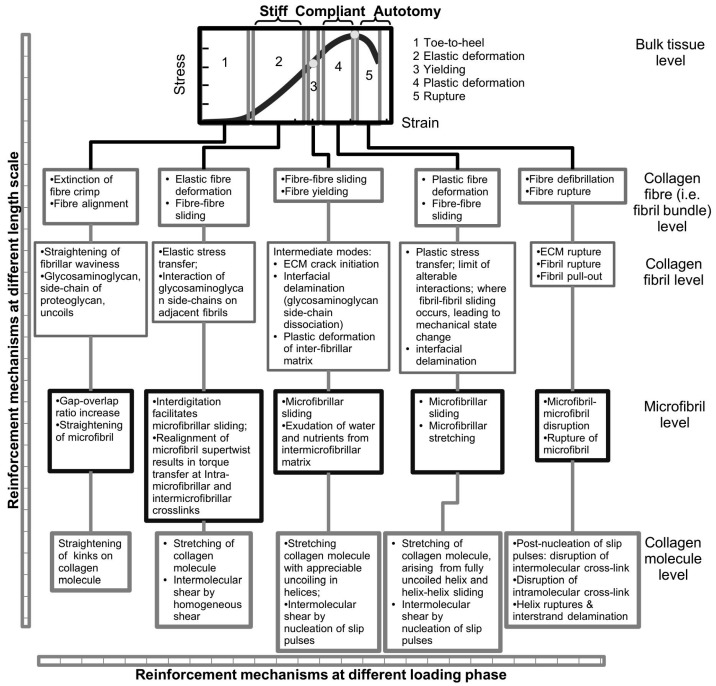
Framework of the ECM mechanics for the MCT [27]. The framework provides a systematic approach to map the mechanisms involve in regulating the mechanical response of ECM at the respective loading regimes, labelled 1–5. These mechanisms are identified across the length scale from molecular to bulk tissue level. At the tissue level, the graph illustrates a schematic representation of typical MCT stress-strain behaviour.

From the practical engineering perspective, currently one of the challenges for the applicability of ECM-DT in tissue engineering is the development of effective decellularization techniques for the removal of cellular components, to minimize immunogenicity upon implantation [35,36,39]. On-going studies to develop effective techniques usually address a combination of physical, chemical and enzymatic methods [174]: physical treatments using cyclical freeze-thawing and ionic solutions can lyse cell membranes, before the enzymatic methods are applied to separate the cellular components from the ECM. Clearly, these processes must be optimized to address high decellularization with minimal effects on the biochemistry and, in particular, the mechanical properties of the ECM. If the optimization approach entails imperfections in the final product, which hierarchical levels (with respect to the framework, Figure 15) can we afford to compromise? The mutability property may be mimicked by incorporating a mechanism to trigger chemicals to cause stiffening or compliance, but this assumes that the decellularization technique will not destroy the other properties related to performing rapid change of mechanical states in the ECM-DT. Further discussion is out of the scope of this review but these issues have been targeted for future investigation.

## 6. Conclusions

New demands for a biomaterial, such as the ECM-DT, that can be used to make scaffolds for tissue engineering applications provide timely opportunity to revisit the fundamental issues and explore the new findings derived from ECM studies. There have been many studies of the ECM of the sea urchin ligaments since the work of researchers such as Hidaka [15,16] and Smith [14]. They have investigated the structure and the composition of both the collagen fibril and the interfibrillar matrix of the tissue, as well as the overall tissue mechanical response. There are also many experimental studies, supplemented by analytical modelling and computer modelling, to investigate the basis of reinforcement of the ECM in the soft connective tissue of vertebrates at the fundamental level since the work of researchers such as Bard and Chapman [175]. This review has addressed several of these findings to prompt new insights which can be summarized as follows.

- MCT deformation characteristics resemble those of mammalian tissues.
- Shear models, addressing elastic and plastic stress transfer, explain the mechanism of collagen fibril reinforcement of MCT during the stiff and compliant states, respectively.
- Nucleation of slip pulses, as a possible mode of collagen fracture, leading to failure of the MCT, could direct autotomy.
- The spindle-like shape in collagen fibrils modulates the stress uptake by ensuring a more uniform distribution of stress throughout the fibril.
- Fibrils with small diameters are responsible for regulating the property of mutability, by addressing the tissue resilience and fracture energy.
- Interplay between the fibril aspect ratio and relative stiffness of collagen to matrix is the key to reducing stress discontinuity in a fibril during fibril-fibril sliding.

## Figures and Tables

**Table 1:** Estimates of the values of the mechanical properties of tissues from the sea urchin.

Tissue	Maximum Stress (MPa)	Stiffness (MPa)	Maximum Strain	Literature
Catch apparatus, sea urchin	40 ^†^	400 ^†^	0.3 ^†^	[15,16]
Tube feet, sea urchin	200 ^#^	2000 ^#^	2.0 ^#^	[70]
Compass depressor, sea urchin	19.5 ± 5.5	40.0 ± 22.3	3.0 ± 2.4	[66]

^†^ indicates that the values are estimates derived from the graphs of stress versus strain. ^#^ indicates that the values are estimates derived from the bar-charts of the respective mechanical properties.

**Table 2:** Examples of the length, diameter and aspect ratio of collagen fibrils in marine invertebrates as well as land vertebrates.

Tissue	Length (*L*_CF_), (μm)	Diameter (2*r*_0_), (nm)	Aspect ratio (*q*)	Literature
**Marine invertebrates**				
Dermis, starfish (*Asterias amurensi*)	196 ^¥^	136 ^¥^	1441 ^‡^	[132]
Dermis, sea cucumber (*Cucumaria frondosa*)	13.8–443.6 ^@^	75 ^#^	184–5914 ^‡^	[133]
Catch apparatus, sea urchin (*Eucidaris tribuloides*)	-	-	2275–3300 ^†^	[22]
Catch apparatus, sea urchin (*Eucidaris tribuloides*)	234 *	75 ^#^	3120 ^‡^	[126]
**Vertebrates**				
Skin, calf (acid-extracted collagen)	9 *	21 ^¥^	423 ^‡^	[134,135]
Tendon, embryonic chick	18 *	50 ^#^	360 ^‡^	[122]
Medial collateral knee ligament, rat	21 *	75 ^#^	282 ^‡^	[120]

^¥^ These are simple averages. ^‡^ These are derived from the ratio of the average length to the average diameter. ^@^ These are broadly observed by the authors of the paper. ^#^ These are estimated from the electron micrographs presented in the paper. ^†^ These are derived from the gradient of a straight line fitted to data points of length versus diameter. * These are estimated values derived from computing the mid-value between the lower and upper limit.

**Table 3:** Estimates of fibrillar and matrix-related Poisson’s ratio and modulus of elasticity parameters for understanding the behaviour of the interfibrillar matrix.

Parameters	Magnitudes	Literature
Poisson’s ratio of collagen fibril, *v*_CF_	2	[156]
Volume fraction of collagen, *V*_CF_	0.2–0.8	[40]
Poisson’s ratio of MCT, *v_c_*	0.7–4.2	[157]
Poisson’s ratio of interfibrillar matrix, *v_m_*	3–18	This review, using Equation (20)
*E*_CF_/*E_m_*	10^3^–10^6^	[80,112]

**Table 4:** Estimates of interfibrillar shear stress.

Interfibrillar Shear Stress	Magnitude (MPa)	Literature
Shear stress, τ_β_, generated at the fibril/matrix interface during the mode β transition stage	1–10	[58]
Shear stress, τ_RP_, generated at the fibril/matrix interface during the fibril rupture or pull-out	100	[58]
Shear stress, τ_GAG_, for rupturing the bonds between proteoglycan glycosaminoglycans using optical tweezers	7.5	[58,166]
Interfibrillar shear stress, τ, by notched tissue testing	32	[109]

## Abbreviations

ACh	acetylcholine
ASW	Artificial sea water
CA	Catch apparatus
ECM	Extracellular matrix
ECM-DT	Extracellular matrix derived from decellularized tissue
EGTA	Ethylene-*bis*-(oxyethylenenitrilo)-tetraacetic acid
FACIT	Fibril associated collagens with interrupted triple helices
MCT	Mutable collagenous tissue
POI	Plane of interest
RVE	Representative volume element
TX	Triton X100

## Non-Greek Symbols

*A*_CF_	Cross-sectional area of the (i.e., uniform cylindrical) fibril
C	C-terminus (containing the carboxyl group) of the collagen molecule
COMP	Cartilage oligomeric matrix protein
*D*	The *D* period of the collagen fibril
*D*_D1_	Population of fibrils with smaller diameter (relative to *D*_D2_)
*D*_D2_	Population of fibrils with larger diameter (relative to *D*_D1_)
*E*_CF_	Tensile stiffness of the collagen
*F*	Axial force generated within the collagen molecule
*F*_max_	Breaking force of the collagen molecule
*G_m_*	Shear modulus of the interfibrillar matrix
*H*	Constant in the differential equation of the shear-lag model
*L*_crit_	Critical length for fibril fracture
*L*_CF_	Half-length of the fibril
*L*_CL_	Contact length between two adjacent collagen molecule
*L*_TC_	Length of the collagen molecule
*m*_l_	Collagen mass per unit length
*M*	Mass of a collagen molecule
*N*	Number of molecules intersecting a fibril cross-section (through an overlap region)
N	Amino-terminus (containing an amine group) of the collagen molecule
*q*	Fibril aspect ratio
(*r*,θ,*z*)	Radial, azimuthal and axial coordinates, respectively, of the cylindrical polar coordinate system
*r_m_*	Radius of the matrix surrounding the fibril
*r*_0_	Radius of the (uniform cylindrical) fibril
*u*_CF_	Axial displacement of the fibril at that point within the fibril
*u_m_*	Axial displacement of the interfibrillar matrix at the same point if the fibril were not presence
*u_E_*	Strain energy density, relates to the tissue resilience
*u_F_*	Strain energy density, relates to the tissue rupture
*v_c_*	Poisson’s ratio of the tissue
*v*_CF_	Poisson’s ratio of the collagen fibril
*v_m_*	Poisson’s ratio of the interfibrillar matrix
*V*_CF_	Volume fraction of collagen fibrils
*V_m_*	Volume fraction of interfibrillar matrix
*Z*	Normalized axial coordinate

## Greek Symbols

α_TC_	Molecular cross-sectional area
β_Cox_	Constant in the shear-lag model; appears in the argument of the trigonometrical functions
ε	Average strain in the fibril
γ_TC_	Energy required to nucleate a slip pulse
λ	Axial overlap distance between two adjacent fibrils
*ρ*	Centre-to-centre lateral separation distance between two adjacent fibrils
*ρ*_Coll_	Collagen density
σ*_c_*	Stress acting on the tissue in the direction of the fibril
σ_Grif_	Applied tensile stress leading to the rupture of the MCT
σ_TC_	Stress associated with *F*
σ*_z_*	Collagen fibril axial stress
τ_β_	Shear stress at the fibril-matrix interface, generated during the mode β transition stage
τ_GAG_	Shear stress for rupturing the bonds between proteoglycan glycosaminoglycans
τ_RP_	Shear stress at the fibril-matrix interface, generated during the fibril rupture or pull-out
τ	Shear stress at the fibril-matrix interface
τ_TC_	Shear resistance between the two collagen molecules
η	Ratio of *L*_CL_ to *L*_TC_
χ_S_	First critical molecular length
χ_R_	Second critical molecular length
